# G3BP1-linked mRNA partitioning supports selective protein synthesis in response to oxidative stress

**DOI:** 10.1093/nar/gkaa376

**Published:** 2020-05-14

**Authors:** Syam Prakash Somasekharan, Fan Zhang, Neetu Saxena, Jia Ni Huang, I-Chih Kuo, Caitlin Low, Robert Bell, Hans Adomat, Nikolay Stoynov, Leonard Foster, Martin Gleave, Poul H Sorensen

**Affiliations:** Vancouver Prostate Centre, Vancouver, BC, Canada; Vancouver Prostate Centre, Vancouver, BC, Canada; Vancouver Prostate Centre, Vancouver, BC, Canada; Vancouver Prostate Centre, Vancouver, BC, Canada; Vancouver Prostate Centre, Vancouver, BC, Canada; Vancouver Prostate Centre, Vancouver, BC, Canada; Vancouver Prostate Centre, Vancouver, BC, Canada; Vancouver Prostate Centre, Vancouver, BC, Canada; Centre for High-Throughput Biology, University of British Columbia, Vancouver, BC, Canada; Centre for High-Throughput Biology, University of British Columbia, Vancouver, BC, Canada; Vancouver Prostate Centre, Vancouver, BC, Canada; Vancouver Prostate Centre, Vancouver, BC, Canada; Department of Molecular Oncology, BC Cancer Research Centre, Vancouver, BC, Canada

## Abstract

Cells limit energy-consuming mRNA translation during stress to maintain metabolic homeostasis. Sequestration of mRNAs by RNA binding proteins (RBPs) into RNA granules reduces their translation, but it remains unclear whether RBPs also function in partitioning of specific transcripts to polysomes (PSs) to guide selective translation and stress adaptation in cancer. To study transcript partitioning under cell stress, we catalogued mRNAs enriched in prostate carcinoma PC-3 cell PSs, as defined by polysome fractionation and RNA sequencing (RNAseq), and compared them to mRNAs complexed with the known SG-nucleator protein, G3BP1, as defined by spatially-restricted enzymatic tagging and RNAseq. By comparing these compartments before and after short-term arsenite-induced oxidative stress, we identified three major categories of transcripts, namely those that were G3BP1-associated and PS-depleted, G3BP1-dissociated and PS-enriched, and G3BP1-associated but also PS-enriched. Oxidative stress profoundly altered the partitioning of transcripts between these compartments. Under arsenite stress, G3BP1-associated and PS-depleted transcripts correlated with reduced expression of encoded mitochondrial proteins, PS-enriched transcripts that disassociated from G3BP1 encoded cell cycle and cytoprotective proteins whose expression increased, while transcripts that were both G3BP1-associated and PS-enriched encoded proteins involved in diverse stress response pathways. Therefore, G3BP1 guides transcript partitioning to reprogram mRNA translation and support stress adaptation.

## INTRODUCTION

Translation of mRNAs is tightly controlled in response to cellular stress, primarily at the initiation step (1). Under diverse forms of cell stress such as oxidative stress, hypoxia, nutrient deprivation, radiation and viral infections, translation initiation is rapidly blocked to limit energy-demanding protein synthesis. This occurs in part through the stress specific eukaryotic initiation factor eIF2α kinases, PKR, PERK, HRI or GCN2, which become activated and phosphorylate eIF2α eIF2·GTP·Met-tRNAMet ternary complexes to block translation initiation and limit global protein synthesis (2). As a result, translationaly stalled mRNAs along with associated 40S ribosomes, RBPs, and translation initiation factors, aggregate in the cytoplasm as translationally inactive mRNA–protein complexes (mRNPs). These mRNPs then transition into highly specialized cytoplasmic structures known as stress granules (SGs) by secondary and tertiary aggregation (2–6). SG nucleation in most cell types requires G3BP1 or its isoform, G3BP2, which shows a more limited expression pattern. G3BP1 is a pleiotropic protein with diverse biological functions (7,8). Apart from its role as a major SG nucleating protein (9), G3BP1 localizes to mitochondria (10–12), endosomes (13) and nucleus (14), where it has largely unknown functions. G3BP1 contains low-complexity (LC), or intrinsically disordered (ID), regions necessary for dimerization (15,16), underlying its ability to function as a nucleating factor for SG assembly. Knockdown (kd) of G3BP1 severely impairs SG assembly in many cell types under arsenite-induced oxidative stress (15,16). Moreover, G3BP1 overexpression alone is sufficient to induce SG nucleation even in the absence of stress (15,17). Other SG nucleating proteins, or proteins critical for SG formation, are also described, such as TIA1 (17) and UBAP2L (18). Like G3BP1, TIA1 kd reduces SG formation and its overexpression drives SG assembly in the absence of stress (17). UBAP2L overexpression nucleates SGs in unstressed cells and UBAP2L is required for both SG assembly and disassembly (19).

Previously, we found a link between G3BP1, SGs and tumor progression. The highly conserved cold-shock domain containing YB-1 protein directly binds to and translationally activates the 5′-untranslated region (UTR) of G3BP1 mRNAs, thereby controlling availability of G3BP1 for SG assembly. YB-1 inactivation in human sarcoma cells dramatically reduced G3BP1 levels and SG formation, and G3BP1 inactivation in sarcoma xenografts prevented *in vivo* SG formation, local tumor invasion, and lung metastasis in mouse models (20). Moreover, elevated G3BP1 expression correlates with poor survival in human sarcomas, where YB-1 and G3BP1 expression is tightly associated. These data highlight novel roles for SG proteins such as G3BP1 and YB-1 in cell survival, adaptation and tumor progression.

Storage of mRNAs in SGs blocks their degradation and allows cells to rapidly restore synthesis of vital proteins encoded by SG-silenced mRNAs during recovery from cell stress, when SGs disassemble (21). In contrast, some mRNAs are known to be excluded from SGs during stress, such as those encoding chaperones and cell damage repair enzymes, possibly supporting continued translation within polysomes (PSs) to facilitate cell viability during acute stress (22–25). Therefore, whether mRNAs reside in SGs or PSs can theoretically play a major role in reprogramming mRNA translation under adverse conditions to facilitate cytoprotective and adaptive responses (26). While the protein and RNA contents of SGs have recently been characterised (9,27–31), much less is known regarding stress-induced partitioning of transcripts between specific SG-associated RBPs such as G3BP1 and polysomes, and how this affects selective translation and stress adaptation.

In the current study, we sought to identify G3BP1-associated transcripts and their partitioning to PSs under oxidative stress. We hypothesize that such partitioning plays a key role for translational reprogramming required for stress adaptation. To test this, we analysed transcripts that are enriched in or depleted from PSs under arsenite-induced oxidative stress using sucrose gradient polysomal fractionation (SGPF) and RNAseq (32,33). In parallel, we profiled transcripts and proteins interacting with G3BP1 under the same conditions, using APEX soybean peroxidase-based proximity-labelling approach, followed by RNAseq (27,34). This revealed that short-term oxidative stress profoundly effects mRNA translation by promoting selective enrichment of some transcripts in PSs, leading to their active translation, while depleting other transcripts from PSs to suppress their translation, and that mRNA association with G3BP1 plays an important role in this compartmentalisation process.

## MATERIALS AND METHODS

### Cell line, antibodies and reagents

PC-3 cells were obtained from ATCC and cultured in DMEM supplemented with 10% FBS. The following antibodies were used: BIOTIN (Cat. 5597) and GAPDH (Cat. 2118) were from Cell Signaling; CLU (Cat. 05-354) and ACTIN (Cat. MAB1501) were from Millipore; FOS (Cat. sc-166940), TIA-1 (sc-1751) and HSPA1A (cat. sc-32239) were from Santa Cruz Biotech; JUN (Cat. 610326) and G3BP1 (Cat. 611127BD) were from BD Biosciences; BAX (Cat. ab32503, ab77566), APX2 (Cat. ab222414) and YB-1 (Cat. ab76149) were from Abcam; HIF1A (Cat. NB100-131) was from Novus; Fluorescently labeled secondary antibodies (mouse, Alexa Fluor 488/594; rabbit, Alexa Fluor 488/594; and goat, Alexa Fluor 488/594), TRIzol, RNAiMAX transfection reagent, Dynabeads M-280 Streptavidin, DMEM, FBS, Click-iT Protein Reaction Buffer Kit, biotin-alkyne and l-azidohomoalanine (AHA) were from Life Technologies; Trolox, biotin-tyramide, sodium ascorbate, sodium deoxycholate and cycloheximide were from Sigma; DMEM without l-lysine and l-arginine was from Caisson Labs (USA); ^13^C_6_-arginine and D4-lysine were from Silantes; FluorSave was from Merck; and G3BP1-APEX and CTRL-APEX constructs were custom made from GenScript.

### Extraction of polysomal transcripts

PSs were purified using a protocol described previously (35). Briefly, two 15 cm PC-3 cells were grown to ∼70% confluency in DMEM + 10% FBS. The cells were then left vehicle treated or treated with arsenite (200 uM) for 2 h. The media was aspirated and replaced by PBS + 100 μg/ml cycloheximide and incubated at 37°C for 10 min. Each dish was then placed on ice, media aspirated, and replaced by ice cold PBS + 100 μg/ml cycloheximide. Cells were scraped, pelleted at 16 000 × g for 30 s, and re-suspended in three pellet-volumes ice-cold hypotonic lysis buffer (10 mM HEPES pH 7.9, 1.5 mM MgCl_2_, 10 mM KCl, 0.5 mM DTT, 1% Triton X-100 and 100 μg/ml cycloheximide). After 10 min, cells were lysed on ice by ten strokes through a 26-gauge needle and nuclei were pelleted at 1500 × g for 5 min. Lysate from ∼15 million cells (one dish) was layered on top of triplicate 10–50% (w/v) sucrose gradients (20 mM HEPES:KOH pH 7.6, 100 mM KCl, 5 mM MgCl_2_, 1 mM DTT and 100 μg/ml cycloheximide) made using a Biocomp Instruments (Canada) gradient master. Gradients were centrifuged for 2 h at 36 000 RPM in a SW-41 rotor, punctured, and manually peak fractionated using real-time A260 monitoring with a Brandel (Gaithersburg, MD) gradient fractionator and ISCO (Lincoln, NE, USA) UA-6 detector. RNA was extracted from pooled technical triplicate sucrose gradient fractions by TRIzol method (Life Technologies, Grand Island, NY), isopropanol precipitated and dissolved in RNase free water.

### Extraction of G3BP1-associated transcripts and proteins

Extraction of G3BP1 associated transcripts was conducted using APEX-based proximity tagging described previously with modifications (18,34). APEX soybean peroxidase was fused in-frame to G3BP1 to generate APEX-G3BP1. Then the empty vector or APEX-G3BP1 were transiently expressed in PC-3 cells. Cells were then exposed for 2 h to −/+ ARS (200 uM) stress. The cells were then pulsed with biotin-tyramide (500 uM for 30 min) and H_2_O_2_ (1 mM for 1 min) to transiently activate the APEX enzyme to link biotin to proteins in close proximity (within 10–20 nm) of the APEX-G3BP1 fusion. The cells were immediately washed 2 times with quencher solution (PBS supplemented with 5 mM Trolox, 10 mM sodium azide and 10 mM sodium ascorbate). The cells were then lysed in lysis buffer (50 mM Tris, 150 mM NaCl, 5 mM Trolox, 10 mM sodium azide, 10 mM sodium ascorbate, 0.1% SDS, 0.5% sodium deoxycholate, 1% Triton X-100 and Protease + phosphatase inhibitors). Clarified the lysate by centrifugation at 13 000 rpm for 10 min at 4°C. Saved small volume of lysate for western blotting. The lysates were then incubated with Dynabeads M-280 Streptavidin to pulldown the biotinylated proteins, and the transcripts were extracted from the pulldowns using TRIzol and processed for RNAseq. For the extraction of G3BP1 associated proteins, a similar experiment was conducted as described above except that the cells were cultured in SILAC media. Cells growing in light amino acid containing media (L) were transfected with CTRL-APEX and cells growing in heavy amino acid containing media (H) were transfected with G3BP1-APEX. The cells were vehicle treated or treated with arsenite (200 uM) for 2 h and subjected to biotin tagging and pulldown as described above. Extracted proteins from vehicle treated or arsenite treated CTRL-APEX and G3BP1-APEX conditions were mixed together, and the processed samples were subjected to mass spec analysis using Orbitrap as described before (36). For the validation of G3BP1 (endogenous) associated transcripts, PC-3 cells were vehicle treated or treated with arsenite as described above. The cells were then UV-cross linked (a short pulse, which favors crosslinking to mRNA rather than proteins) and subjected to riboimmunoprecipitation using anti-G3BP1 antibodies. The immunoprecipitated samples were washed thoroughly and subjected to qRT-PCR.

### RNA sequencing

Transcripts extracted from PSs and G3BP1 complexes were depleted of the ribosomal RNA using Ribo-Zero (Illumina, San Diego, CA, USA) and the sample quality was evaluated using the Agilent 2100 Bioanalyzer. Qualifying samples were then prepped following the standard protocol for the NEBNext Ultra II Stranded mRNA (New England Biolabs). Sequencing was performed on the Illumina NextSeq 500 with Paired End 42 bp × 42 bp reads. De-multiplexed read sequences were then aligned to the *Homo sapiens* UCSC hg19 reference sequence using STAR (37) aligner. Assembly and differential expression were estimated using Cufflinks (http://cole-trapnell-lab.github.io/cufflinks/) through bioinformatics apps available on Illumina Sequence Hub.

### Software used for data analysis

Volcano plots were constructed using Microsoft Excel software. Venn diagrams were created using VENNY program available online (https://bioinfogp.cnb.csic.es/tools/venny/). Gene ontology analysis was performed using Metascape software available online (http://metascape.org). The genes were uploaded in the website and express analysis option was selected as the desired form of analysis. Metascape automatically first converted the input identifiers (Gene Symbol) into Human Entrez Gene IDs. The software then identified all statistically enriched terms such as GO/KEGG terms, canonical pathways and hall mark gene sets, and accumulative hypergeometric *P*-values and enrichment factors were calculated and used for filtering. Subsequently, the remaining significant terms were then hierarchically clustered into a tree based on kappa-statistical similarities among their gene memberships. Then 0.3 kappa score was applied as the threshold to cast the tree into term clusters.

### Immunoblotting

PC3 cells transfected with siControl or siG3BP1 siRNAs were treated with 200 uM arsenite for different time points (1–8 h). Cells were gently scraped off from the culture dishes with a cell scraper, washed with PBS, and lysed using lysis buffer (20 mM Tris–HCl, pH 7.5, 150 mM NaCl, 1 mM Na_2_EDTA, 1 mM EGTA, 1% Triton X-100 and 1× protease inhibitor). Cell lysates were centrifuged at 5000 rpm for 10 min and the supernatant was saved. Protein concentration was determined using a Bradford assay (Bio-Rad Laboratories). Protein lysates were mixed with 2× loading dye, and equal amount of proteins were separated in 4–12% gradient SDS-PAGE and immunoblotted into nitrocellulose membrane using wet transfer as described previously (38). For the analysis of newly synthesized proteins, PC-3 cells vehicle treated or treated with arsenite were incubated with 50 uM AHA (Azidohomoalanine) for 1 h. The cells were then lysed, the newly synthesized proteins were derivatized with alkyne biotin. The biotinylated proteins were pulled down using Streptavidin beds and subjected to immunoblotting for anti-biotin antibodies as described before (36).

### Quantitative RT-PCR

qRT-PCR was performed as described before (20). Polysomal, total and G3BP1-associated transcripts were reverse transcribed to cDNA. The cDNA was subjected to qRT-PCR using oligo pairs for genes provided in Supplementary Table S9.

### Extracellular flux analysis

Glycolysis stress test kit (Agilent technologies, 103020-100) was used to measure extracellular acidification rate (ECAR) and oxygen consumption rate (OCR). Briefly, PC3 cells were plated at 4000 cells per well cell density in XF96 well plate pretreated with poly-lysine in RPMI plus FBS media. On the day of experiment, media was replaced with phenol-red free Seahorse XF base medium (Agilent, 103335-100) containing 2 mM l-glutamine. The cells were treated with 200 μM arsenite for 3 h. 10 mM glucose, 2 μM oligomycin and 50 mM 2-DG were added in ports A, B and C on the indicated times. Data was normalized to cell number measured by crystal violet assay.

### ATP measurement

PC3 cells were plated at 4000 cells per well cell density in 96-well plate in RPMI plus FBS media followed by treatment with 200 μM arsenite for 3 h. ATP assay was performed as per manufacturer's guidelines (Abcam, ab113849).

### Cell cycle analysis

Cell cycle analysis was performed as described before (39). Briefly, PC-3 cells were vehicle treated or treated with arsenite. The cells were harvested, washed with PBS and fixed in cold 70% ethanol. The cells were again washed and treated with 50 μl of a 100 μg/ml stock of RNase. The cells were then incubated with 200 μl PI (from 50 μg/ml stock solution), and analysed by FACS.

### *In situ* hybridization and immunofluorescence microscopy

Cells seeded at 20–25% confluence in 6-cm culture dishes containing round cover glasses (12CIR-1D; Thermo Fisher Scientific) were treated with vehicle alone or exposed to arsenite stress (200 uM for 2 h). Immunofluorescence (IF) was performed as described previously (20). Cells were fixed in 4% paraformaldehyde (PAF) for 20 min and permeabilized with PBS-T (0.05% Triton X-100 in PBS) for 20 min. The cells were then blocked for 30 min in PBS-T containing 5% BSA and incubated with primary antibodies (1:100) for 1 h in PBS containing 2.5% BSA. Cells were washed in PBS-T for 30 min (3 × 10 min) followed by incubation with secondary antibodies (1:200) in PBS-T containing 2.5% BSA for 1 h. Cells were then washed in PBS-T for 30 min (3 × 10 min). For the localization of mRNA, manual *in situ* hybridization (ISH) was performed as described previously (20,40) using fluorescent labelled probes targeting *BAX, FOS and HSP70*, purchased from Integrated DNA Technologies (see Supplementary Table S9 for the sequences of probes used) or chromogenic probes targeting *CDKN3* (Cat. 401621), *EIF4EBP1* (Cat. 456861), *HIF1A* (Cat. 605221) and *BAX* (Cat. 573661), purchased from Advanced Cell Diagnostics. For ISH using fluorescent probes, coverslips containing cells were incubated in hybridization buffer (2× SSC, 20% formamide, 0.2% BSA, and 1 μg/μl yeast tRNA) for 15 min at 37°C. Subsequently, cells were hybridized with fluorescent-labelled hybridisation probes at 37°C. After 24 h of incubation, cells were washed twice with 2× SSC and 20% formamide for 5 min at 37°C, twice with 2 × SSC for 5 min each at 37°C, and once with 1× SSC for 5 min at 37°C. Manual chromogenic assays were performed using probes using protocols supplied by Advanced Cell Diagnostics. The ISH slides were then subjected to immunostaining with anti-G3BP1 antibodies as described above. All the cells processed as in this paragraph were immersed in DRAQ5 (10 μM; Biostatus) for nuclear staining, mounted with FluorSave, and viewed using an inverted confocal microscope (Eclipse Ti-E; Nikon) with 40× and 100× oil-immersion objective lenses. Images were captured using EZ-C1 software and were further processed using ImageJ software.

## RESULTS

### Identification of polysome-enriched versus -depleted transcripts under arsenite stress

**Figure 1.**
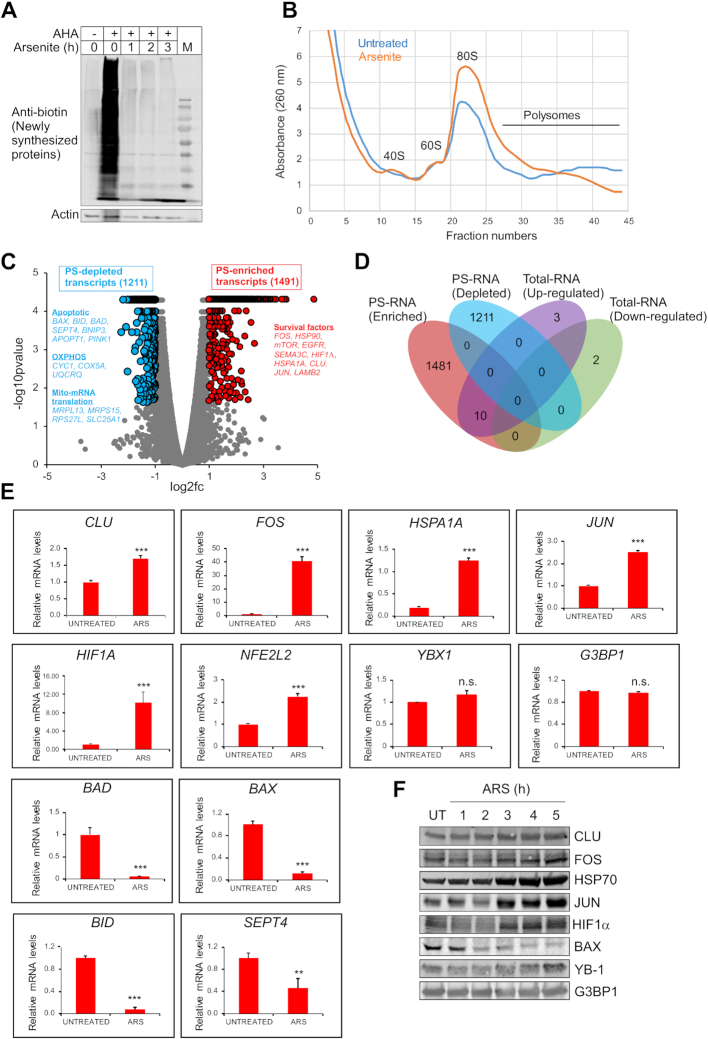
Isolation of PS-enriched and PS-depleted transcripts and their validation. (**A**) Detection of newly synthesized proteins in untreated or arsenite treated cells using AHA (azidohomoalanine)-mediated CLICK method (36). Cells cultured in AHA-free medium functioned as the control. Note that arsenite stress reduced the synthesis of new proteins. (**B**) Polysomal trace of untreated or arsenite treated cells. (**C**) Volcano plot showing PS- enriched or PS-depleted transcripts in response to arsenite treatment. Blue balls on the left depict PS-depleted transcripts (log_2_fold −1.0 and below, *P* value < 0.05), with name of a few transcripts and corresponding pathways are shown in blue. Red balls on the right depict PS-enriched transcripts (log_2_fold 1.0 and above; *P* value < 0.05), with name of a few transcripts and corresponding pathways are shown in red. Grey balls represent unchanged/non-significant transcripts. (**D**) Venn diagram comparing PSseq data and total transcriptome data. (**E**) Validation of PSseq data for selected transcripts by qRT-PCR using polysomal RNA extracted from vehicle-treated/untreated (UT) or arsenite-treated (ARS) cells. Mean values ± SD are shown for three independent experiments. ***P < 0.001; **P < 0.01; ns, non-significant. (**F**) Validation of PSseq data of selected transcripts using Western blotting of untreated/vehicle treated (UT) or arsenite-treated cells.

As a first step to explore the effects of oxidative stress on global translation, we analysed new protein synthesis in response to arsenite treatment in PC-3 prostate cancer (PCA) cells, using Click-chemistry based azidohomoalanine (AHA) labelling as described (36). As expected based on the literature (41), arsenite treatment significantly reduced global protein synthesis (Figure 1A). To probe selective mRNA translation under oxidative stress, we catalogued transcripts associated with PSs under arsenite treatment (2 h at 200 uM). PS-associated transcripts were isolated from arsenite (ARS)- versus untreated/vehicle-treated (UT) PC-3 cells using SGPF (Sucrose Gradient Polysomal Fractionation) (see polysome profiles in Figure 1B) and subjected to RNAseq (henceforth termed PSseq) from triplicate samples, as described (32). PSseq was normalised and differential expression of PS-associated transcripts was assessed with established methods such as cufflinks (42,43), available at Illumina BaseSpace-sequence-hub (https://www.illumina.com/products/by-type/informatics-products/basespace-sequence-hub/apps/cufflinks-assembly-de.html). Cufflinks software assembles transcripts, estimates their abundances, and tests for differential expression and regulation in RNASeq samples. It accepts aligned RNASeq reads and assembles them into a parsimonious set of transcripts. Cufflinks then estimates the relative abundances of these transcripts based on how many reads support each transcript, taking into account biases in library preparation protocols (http://cole-trapnell-lab.github.io/cufflinks/). Data is shown as a heat map of differential gene expression from triplicate samples in Supplementary Figure S1A, with correlation analysis in Supplementary Figure S1B. From this analysis we identified 6290 differentially expressed (ΔGene Count) genes, with 3130 demonstrating enrichment and 3159 showing reduced expression (Supplementary Table S1), to which we applied stringent cut-offs to define polysomal transcripts. Transcripts associated with PSs (i.e. PS-enriched) in arsenite stressed cells were defined based on a fold change (FC) of transcript abundance in arsenite-treated versus vehicle treated cells of log2fold 1.0 and above, while transcripts depleted from PSs (i.e. PS-depleted) in arsenite stressed cells were defined based on a FC of log_2_fold -1.0 or less when the same cells were compared. All transcripts selected based on the above cut-offs were statistically significant (*P* value < 0.05). Using these definitions, we identified 1491 PS-enriched and 1211 PS-depleted transcripts (Figure 1C).

Since arsenite may also affect expression of individual mRNAs by altering their transcription or degradation rates, which could indirectly affect their apparent PS association, we isolated and sequenced total RNA from the same cell populations and analyzed it by total RNAseq. The data was normalized as above, and transcripts are listed in Supplementary Table S2. Data is shown as a heat map of differential gene expression from triplicate samples in Supplementary Figure S1C, with correlation analysis in Supplementary Figure S1D. Notably, expression of only a very small fraction (0.67%) of total transcripts was significantly increased (log_2_fold 1 and above) or decreased (log_2_fold −1 or less) at the transcriptional level in arsenite-treated compared to vehicle treated cells. A comparison of PS-enriched and PS-depleted transcripts to total RNA expression of transcripts altered in response to arsenite stress is shown in Figure 1D, highlighting that there was very little overlap among these compartments. Together, our results demonstrate that while short-term oxidative stress induced by arsenite has a negligible effect on total mRNA levels, it has a marked effect on their PS distribution. Raw data for PSseq and total RNAseq were submitted to NCBI GEO at the following URL (https://www.ncbi.nlm.nih.gov/geo/query/acc.cgi?acc=GSE138058).

### Validation and gene ontology analysis of PSseq results

We next validated the above PSseq data using quantitative RT-PCR (qRT-PCR) to determine expression levels of transcripts enriched in PSs in the presence or absence of arsenite stress. PS-enriched transcripts were purified by SGPF from arsenite-stressed versus vehicle alone treated PC-3 cells as above. Transcripts were then reverse transcribed and subjected to qRT-PCR for selected transcripts retrieved from our PSseq data (see Supplementary Table S1). This confirmed that, after arsenite treatment, mRNAs such as *CLU, FOS, HSPA1A (HSP70), JUN, HIF1A* and *NFE2L2* (encoding NRF2) were significantly enriched in PS fractions, while some transcripts such as *G3BP1* and *YBX1* were unchanged, and certain transcripts such as *BAD*, *BAX*, *BID* and *SEPT4* were significantly reduced in PS fractions (Figure 1E). Western blot analysis of PC-3 cells ± arsenite treatment was then used to analyse protein expression of selected transcripts from the PSseq data. Levels of CLU, FOS, HSPA1A (HSP70), JUN and HIF1α, whose mRNAs were enriched in PSs under arsenite stress (Supplementary Table S1), were enhanced in arsenite-treated compared to control cells (Figure 1F). Protein levels of G3BP1 and YB-1, whose mRNAs were unchanged in PSs under arsenite stress, were also relatively constant in arsenite-treated compared to control cells (although YB-1 showed a slight increase at 5 h). Finally, protein levels of BAX, whose mRNA is depleted in PSs under arsenite stress, was also reduced in arsenite-treated compared to control cells (Supplementary Table S1; Figure 1F). To further verify our results, we assessed acute synthesis of proteins encoded by *JUN*, *HSPA1A (HSP70)*, *HIF1A* and *BAX*, using Click chemistry AHA-mediated pulldown of newly synthesized proteins (36). As shown in Supplementary Figure S2A, this showed that that PS-enriched or depleted transcripts are indeed selectively synthesized at enhanced or reduced rates, respectively, under arsenite stress (Supplementary Figure S2A). Collectively, these results serve to validate the observed PSseq data, at least for the tested transcripts.

Next, we compared our data to proteins previously reported to be up- or downregulated in response to arsenite treatment (44–48) and assessed corresponding mRNAs in our PSseq data. Transcripts encoding arsenite-induced upregulated proteins from those studies, including *MT1X, MTHFD1, SELENBP1, HSPA1A (HSP70), SERPINH1, HMOX1, HSPA1B, HSP90, HSPA5, HSPA2, HSPA6, COL6A1, RRBP1, AASS, PC, SEC31A, MTHFD1, PDIA3, OPLAH, HSPH1, SELENBP1, ACO1, SUOX* and *HIF1A*, were also PS-enriched in our study, and transcripts encoding downregulated proteins such as *RANBP1, GPX4, ASNA1, RAB11B, TPM1, ARPC2*, and *AK1*, were also PS-depleted in our study, providing further support for the validity of our PSseq data (Supplementary Table S1).

We also compared our PSseq data with a recent proteomic study highlighting proteins that evade stress-induced translational repression in arsenite-treated cells, as identified by quantitative bio-orthogonal noncanonical amino acid tagging (BONCAT) and stable isotope labeling by amino acids in culture (SILAC) (41). That study revealed hundreds of proteins that remain actively synthesized during stress-induced translational repression in arsenite treated human neuroblastoma cell lines. Out of 362 proteins in their list, 27% (97 proteins) and 68% (245 proteins) of proteins evading translational repression in their set are shared with PS-enriched and PS-unchanged transcript fractions, respectively, while a minor fraction (2%; 9 proteins) is shared with our PS-depleted fraction. Therefore 95% of proteins from Baron *et al.* that evade translational repression after arsenite stress are either enriched or unchanged in their association with PSs after arsenite stress in our studies (Supplementary Figure S2B). These observations further support the robustness of our PSseq data, and reinforce the concept of using PSseq as a translational readout to study newly synthesized proteins under stress.

**Figure 2.**
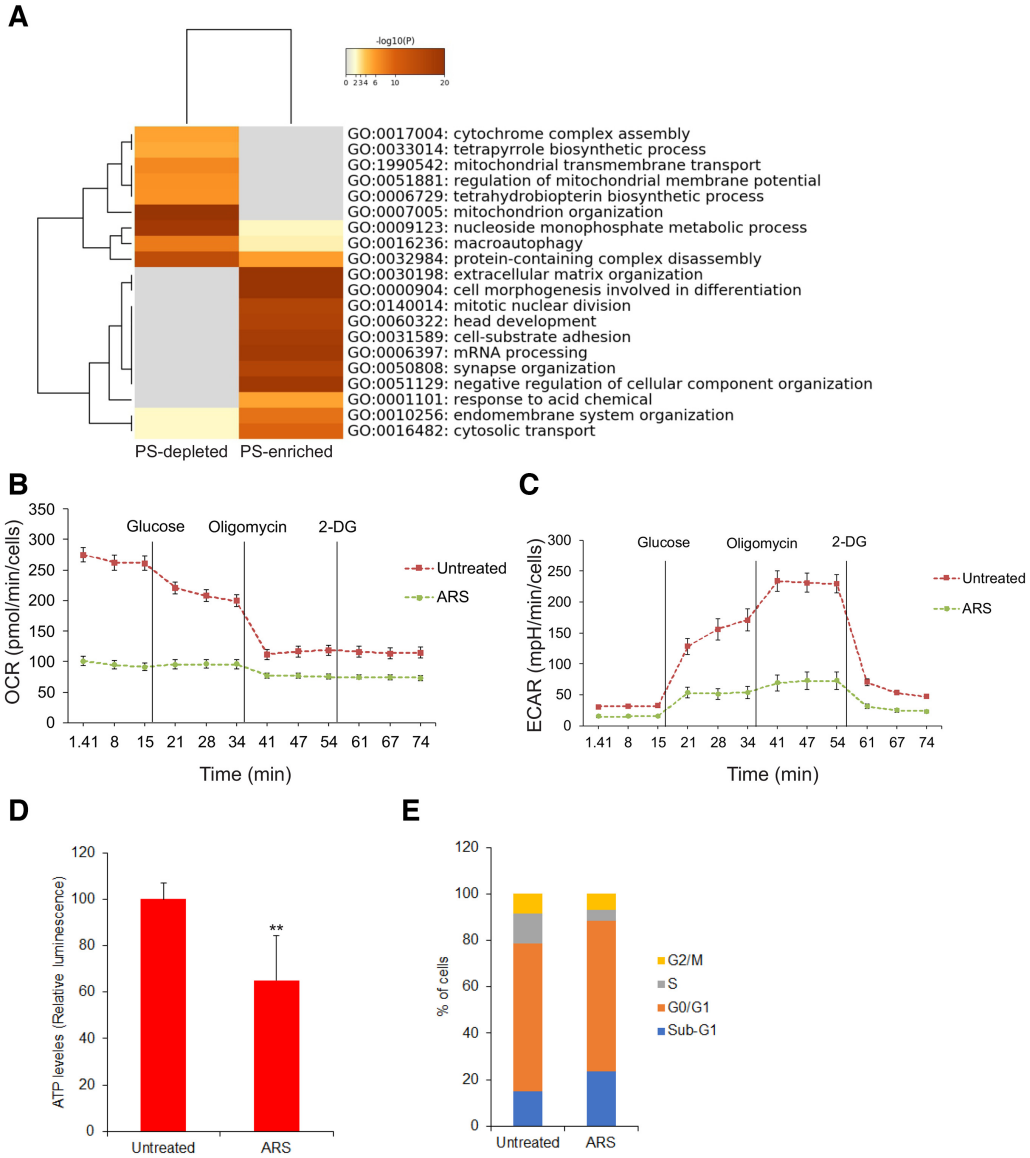
Pathway analysis of PS-enriched and PS-depleted transcripts and functional studies. (**A**) Gene ontology analysis of PS-enriched and PS-depleted transcripts using Metascape software (http://metascape.org). Oxygen consumption rate (OCR) (**B**) and extracellular acidification rate (ECAR) (**C**) were determined through real-time measurements using the Seahorse XF24 Extracellular Flux Analyzer (see Methods for the details). Mean values ± SD are shown for five independent experiments. (**D**) Measurement of ATP in untreated or arsenite treated cells. Mean values ± SD are shown for three independent experiments. **P < 0.01. (**E**) Cell cycle analysis of untreated or arsenite treated cells stained with propidium iodide and analysed by FACS.

Next, Metascape software (http://metascape.org) was used for comparative gene ontology analyses of pathways associated with PS-enriched and PS-depleted transcripts. The top 20 statistically significant pathways generated using this approach are shown in (Figure 2A). Based on this analysis, PS-enriched transcripts are involved in extracellular organisation, cell morphogenesis and differentiation, mitotic nuclear division, head development, cell substrate adhesion, mRNA processing, synapse organisation, negative regulation of cellular component organisation, response to acidic chemicals, endomembrane system organisation, and cytosolic transport. Notably, we observed mRNAs encoding cytoprotective and anti-apoptotic proteins such as FOS, HSP90, mTOR, EGFR, SEMA3C, HIF1α, HSPA1A, CLU, JUN and LAMP2 in the PS-enriched category (Figure 1C; Supplementary Table S1), suggesting that associated pathways may be activated to protect cells during oxidative stress. Proteins encoded by PS-depleted transcripts under stress are linked to mitochondria-related functions such as pro-apoptotic activity (BAX, BID, BAD, SEPT4, BNIP3, APOPT1 and PINK1), mitochondrial membrane potential, cytochrome complex assembly, oxidative phosphorylation (CYC1, ATP5H, COX5A and UQCRQ), and mitochondrial translation (MRPL13, MRPS15, RPS27L and SLC25A1), pointing to translational suppression of mRNAs encoding these proteins, potentially for energy conservation and cell survival (Figure 1C; Supplementary Table S1).

Based on the above ontology analysis, and given that arsenite inhibits mitochondrial function and ATP production (49) during stress adaptation (49–52), we next assessed mitochondrial bioenergetics in the presence of arsenite. We therefore measured ATP levels as well as oxygen consumption rates (OCR) as readouts of oxidative phosphorylation, and extracellular acidification rate (ECAR) as a readout of glycolysis using Seahorse technology. As reported (53), arsenite treatment reduced respiration (Figure 2B**)** and glycolysis (Figure 2C), leading to ATP depletion (Figure 2D), thus functionally validating our ontology analysis. Arsenite has also been shown to delay progression through the cell cycle and to induce apoptosis following G2/M Arrest (54,55). Accordingly, arsenite induced cell cycle arrest, as determined by FACS analysis (Figure 2E), in agreement with our pathway analysis and as reported (56).

### Identification of G3BP1-enriched proteins using APEX-based spatially restricted enzymatic tagging

**Figure 3.**
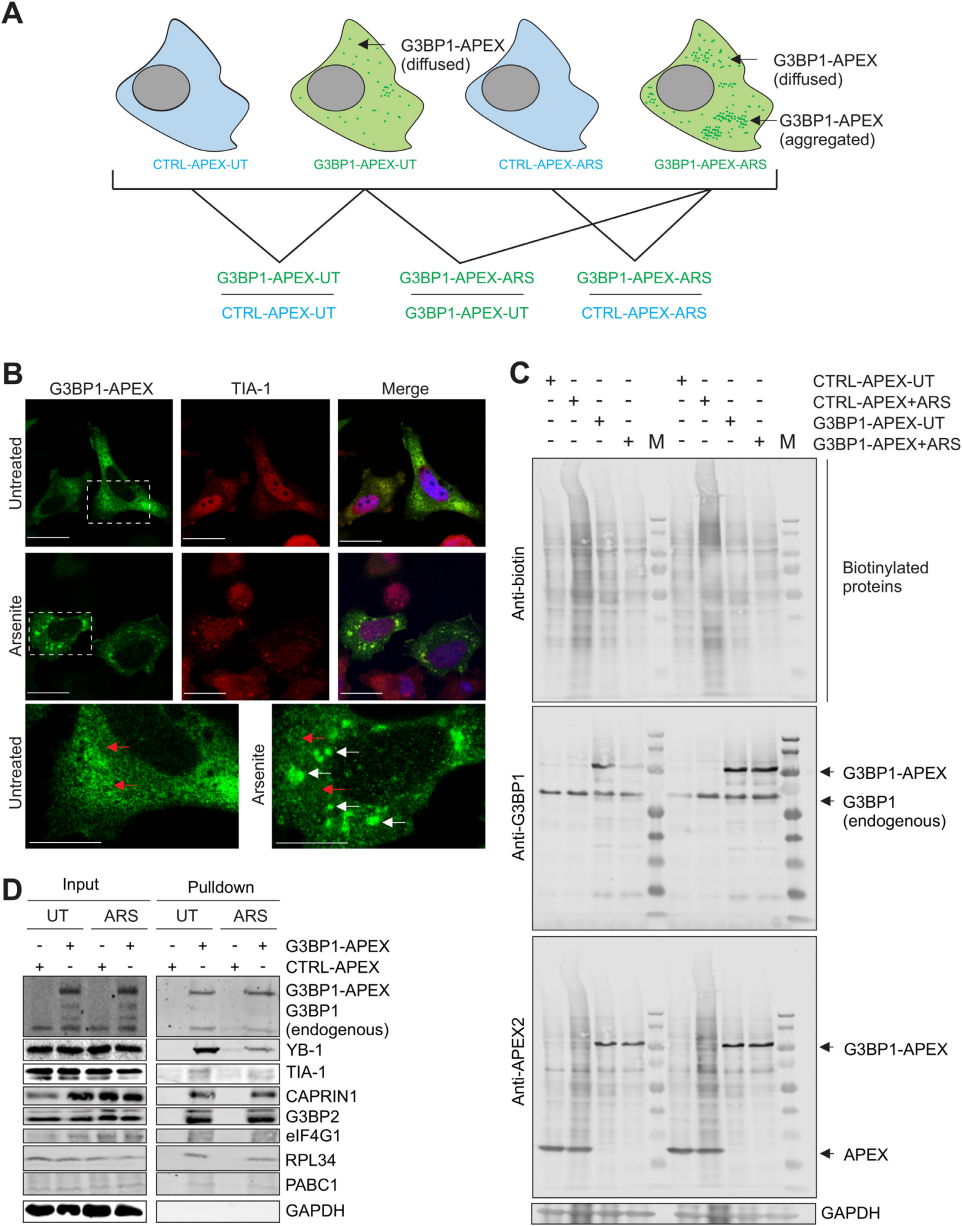
APEX method for the extraction of G3BP1-associated proteins and transcripts. (**A**) Schema for extracting and comparing G3BP1-associated proteins and transcripts. G3BP1-APEX, represented by green dots, is present as diffused in the vehicle treated cells while it is present as both aggregated as well as diffused in the arsenite treated cells. See Methods for details. (**B**) APEX-G3BP1 colocalization by immunofluorescence was assessed using the SG protein, TIA-1. A part of the image of APEX-G3BP1 immunostaining from vehicle treated and arsenite treated cells is enlarged and shown at the bottom panels. Note that G3BP1 is mainly present as diffused in the vehicle treated cells (red arrows) while it is present as both aggregated (white arrows) and diffused (red arrows) in the arsenite treated cells. Scale, 10 um. (**C**) Western blot showing detection of biotinylated proteins (lanes 1–4 and lanes 6–9) as detected using anti-biotin antibodies (first upper panel), G3BP1-APEX fusion protein and G3BP1 (endogenous) as detected using anti-G3BP1 antibodies (second panel), G3BP1-APEX fusion protein and APEX alone as detected by anti-APX2 (APEX2) antibodies (third panel). GAPDH is used as the loading control. (**D**) Western blot analysis to detect proteins associated with APEX-G3BP1 complexes in unstressed and arsenite treated cells.

Next, to determine the role of G3BP1 in partitioning mRNAs to or away from PSs under stress, we isolated G3BP1-interacting proteins and mRNAs in PC-3 cells under different conditions by adapting an APEX-based proximity-labelling (18,34,57). The soybean ascorbate peroxidase APEX2 (APX2) was ligated in-frame with G3BP1 to generate an G3BP1-APEX expression plasmid. G3BP1-APEX or an APEX control (CTRL-APEX) was transiently expressed in PC-3 cells cultured either in conventional medium (for RNAseq) or in SILAC medium (for subsequent mass spectrometry; see Materials and Methods). Cells were then treated with arsenite (ARS) or vehicle (UT) for 2 h to generate four experimental cell populations (CTRL-APEX-UT, CTRL-APEX+ARS, G3BP1-APEX-UT, and G3BP1-APEX+ARS), each prepared in triplicate (Figure 3A). Expression of APEX-G3BP1 was confirmed by immunofluorescence (IF; Figure 3B). G3BP1-APEX showed a predominantly diffuse distribution in the cytoplasm of vehicle treated cells (see red arrows in enlarged image of Figure 3B; vehicle treated panel). In contrast, a proportion of G3BP1-APEX formed distinct aggregates (see white arrows in the enlarged image of Figure 3B; arsenite panel), along with some G3BP1-APEX retaining a diffuse distribution (see red arrows in the same panel). This pattern of differential localisation of G3BP1 is depicted schematically in Figure 3A. Under arsenite treatment, a significant proportion of G3BP1-APEX co-localized with the known SG protein, TIA1 (16) (Figure 3B), in accordance with published data that ∼20% of G3BP1 is enriched in SGs under arsenite stress (9), and confirming that at least a fraction of G3BP1-APEX partitions to SGs under arsenite.

The above cells were incubated with biotin-tyramide for 30 min followed by H_2_O_2_ treatment for 1 min to activate the APEX enzyme and covalently link biotin to intracellular proteins in close proximity (i.e. within 10–20 nm) with G3BP1-APEX, as described (57). To confirm biotinylation, small aliquots of each cell lysate were blotted using anti-biotin antibodies, which detected multiple protein bands (Figure 3C; top panel), confirming that the APEX enzyme is active. Blotting with antibodies to G3BP1 (Figure 3C; second panel) and APEX2 (APX2) (Figure 3C; third panel) detected the G3BP1-APEX fusion protein, endogenous G3BP1 and APEX2 protein alone. Cells were washed and lysed, and G3BP1-enriched fractions were then affinity purified with Streptavidin beads (see Methods). Known SG-associated proteins, YB-1, TIA-1, CAPRIN1, G3BP2, eIF4G1, RPL34 and PABC1, were co-purified in G3BP1-APEX pulldowns, further validating our methodology (Figure 3D).

**Figure 4.**
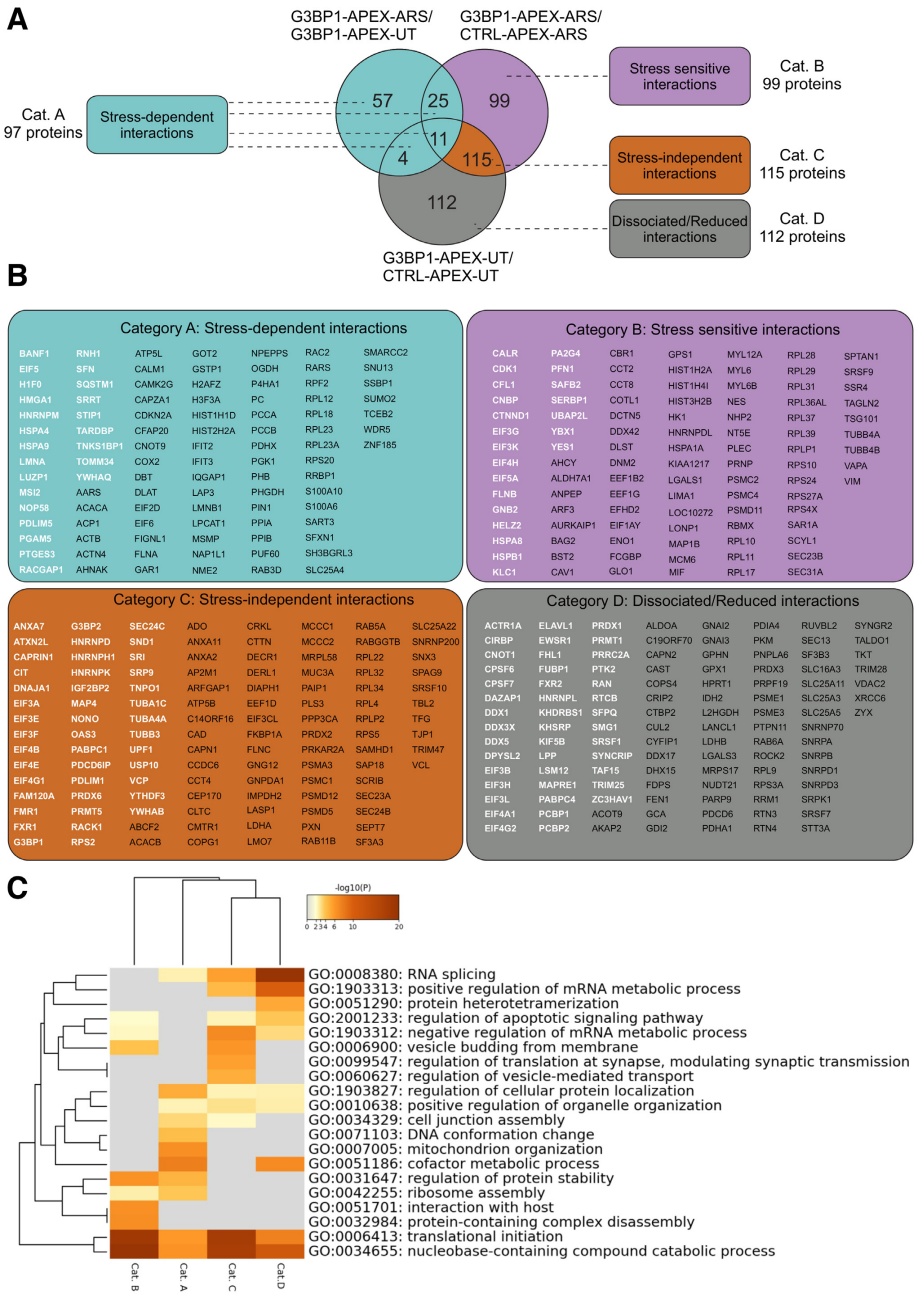
G3BP1-associated proteins and pathway analysis. (**A**) Venn diagram showing a comparison and categorization (4 categories) of G3BP1-associated proteins in unstressed and arsenite stressed cells (see text for more details). (**B**) Proteins that come under the above different categories are listed in boxes with the corresponding colour shades. SG/G3BP1-interacting proteins, already reported in the literature are shown in white letters and new proteins identified in the current study are shown in black letters. (**C**) Gene ontology analysis using the above four categories of proteins using Metascape software (http://metascape.org).

To identify G3BP1-associated proteins, APEX pulldown samples from respective cell populations grown in SILAC medium were processed for LC-MS/MS as described (36). Abundance scores for each protein were averaged from triplicates of three different comparisons, namely G3BP1-APEX-ARS/G3BP1-APEX-UT, G3BP1-APEX-ARS/CTRL-APEX-ARS and G3BP1-APEX-UT/CTRL-APEX-UT (presented as a quantitative matrix in Supplementary Table S3, and deposited on the ProteomeXchange server as dataset identifier PXD015491). Correlation analysis of proteins in the above three comparisons are provided in Supplementary Figures S3A-C. Statistically significant scores (i.e. *P* value < 0.05) were selected and the three conditions were compared in a Venn diagram (Figure 4A). From the latter, we organised the data into four categories of G3BP1-associated proteins, namely interactions that were stress-dependent (green shaded), stress sensitive (purple shaded), stress independent (light brown), and non-associated/reduced (grey shaded) after arsenite stress (Figure 4A). Proteins included in each of the above categories are listed in Figure 4B. Stress-dependent interactions (Figure 4B; Category A; 97 proteins;) showing increased association with G3BP1 only after arsenite stress included known SG proteins such as BANF1, EIF5, PGAM5, RNH1 and TARDBP (TDP-43), while stress sensitive (Category B; 99 proteins), which showed increased binding to G3BP1 after stress, included known SG proteins EIF3G, EIF4H, EIF3K, GNB2, PFN1, SERBP1, UBAP2L and YBX1 (YB-1). The stress independent group (Category C; 115 proteins), composed of proteins associated with G3BP1 irrespective of stress, included known SG proteins such as CAPRIN1, EIF3A, EIF4G1, FMR1, FXR1, G3BP1, G3BP2, HNRNPK, IGF2BP2, RACK1, USP10, VCP and YTHDF3, which also aligns with previous studies suggesting that the majority of proximal and distal interactors of G3BP1 remain the same after stress (18,27). An additional group with reduced G3BP1 interactions after stress (Category D; 112 proteins) included DDX family proteins such as DDX1, DDX3X and DDX5, PCBP proteins including PCBP1 and 2, nucleocytoplasmic shuttling factor SRSF1, and CNOT1, a member of the CCR4-NOT deadenylation complex. The latter was validated experimentally, as the association of CNOT1 with G3BP1 was reduced by arsenite (Supplementary Figure S4A).

We also compared G3BP1 and SG associated proteins from our studies with those in the literature (18,27,28), the RNA granule database (http://rnagranuledb.lunenfeld.ca/), and the SG proteome database (https://msgp.pt/). Overlaps are shown in white letters in Figure 4B, while proteins that are newly identified in our study are shown in black. Furthermore, we selected several G3BP1-associated proteins from our APEX studies and confirmed that they bind to G3BP1, using pulldown of endogenous G3BP1 from PC-3 cells with anti-G3BP1 antibodies. This demonstrated that endogenous G3BP1 indeed binds to YB-1, TIA1, CAPRIN1, G3BP2, and eIF4G (Supplementary Figure S4B), further supporting our studies. Gene ontology analysis was then used to determine pathways linked to observed G3BP1 interacting proteins for categories A-D of Figure 4A. The top 20 enriched pathways are shown in Figure 4C, based on *P* values <0.05 by Metascape analysis, demonstrating that G3BP1 interactors are involved in diverse biological processes, including translation (e.g. RNA splicing, translation initiation, and ribosome assembly).

### Identification of G3BP1-enriched versus G3BP1-depleted transcripts under oxidative stress

**Figure 5.**
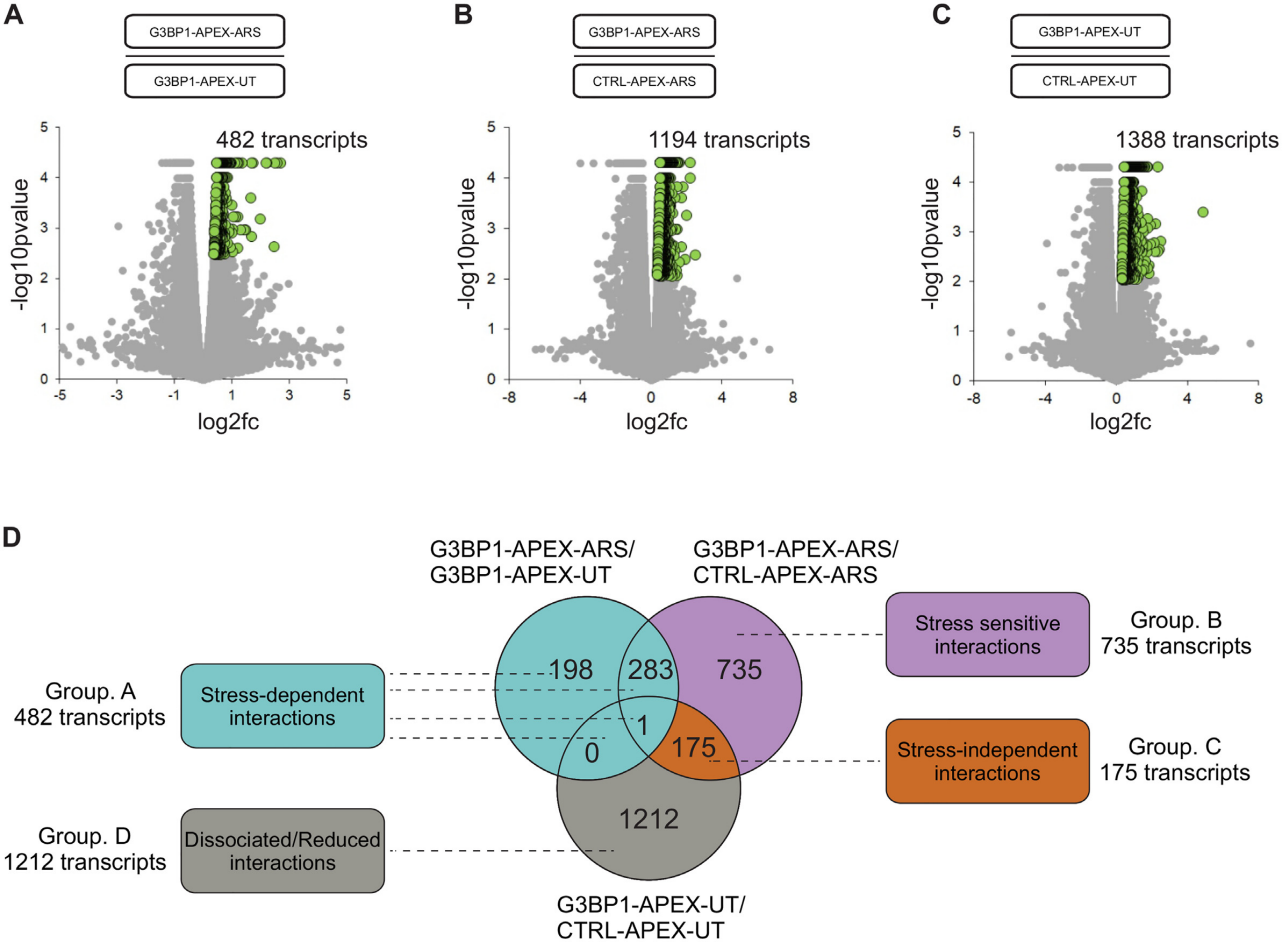
RNAseq of G3BP1 associated transcripts. Volcano plot showing G3BP1 associated transcripts with comparisons: (**A**) G3BP1-APEX-ARS/G3BP1-APEX-UT; (**B**) G3BP1-APEX-ARS/CTRL-APEX-ARS and (**C**) G3BP1-APEX-UT/CTRL-APEX-UT (see text for more details). (**D**) Venn diagram showing a comparison of G3BP1-associated transcripts in unstressed and arsenite stressed, dividing the transcripts into four groups, namely stress-dependent (green shaded), stress-sensitive (purple shaded), stress-independent (light-brown shaded), and transcripts with reduced or dissociated interactions (grey shaded) after arsenite stress.

To catalogue transcripts associated with G3BP1, we extracted polyA RNA from triplicate samples of the above biotinylated G3BP1 complexes. These were used to prepare cDNA libraries, which were then subjected to whole transcriptome RNAseq, as described (37). We performed three separate comparisons to identify G3BP1-associated transcripts, using a similar strategy as described above for G3BP1 protein interactors. First, we catalogued differential G3BP1-associated transcript abundance in G3BP1-APEX-ARS versus G3BP1-APEX-UT cells, to identify transcripts binding to G3BP1 under arsenite treatment. A heat map showing transcript abundance is shown in Supplementary Figure S5A, and corresponding correlation analysis in Supplementary Figure S5B. This identified 482 G3BP1-associated transcripts under arsenite stress (i.e. having a fold-change of log2fold 0.37 or more in transcript abundance in G3BP1-APEX-ARS versus G3BP1-APEX-UT cells and with *P* < 0.05; Figure 5A; Supplementary Table S4). Second, we compared differential transcript abundance in G3BP1-APEX versus CTRL-APEX expressing cells to control for transcripts associated with G3BP1 rather than APEX, and in G3BP1-APEX-UT versus CTRL-APEX-UT cells to identify transcripts associated with G3BP1 under non-stress conditions. Corresponding heat maps and correlation analyses for the latter two comparisons are shown in Supplementary Figures S5C and S5D, and Supplementary Figures S5E and S5F, respectively. This identified 1194 G3BP1-associated transcripts under arsenite stress (Figure 5B; Supplementary Table S5) and 1388 G3BP1-associated transcripts under non-stress conditions (Figure 5C; Supplementary Table S6), each defined as having a fold-change of log_2_fold 0.35 or more (*P* value < 0.05) in transcript abundance in their respective comparisons.

Using a similar strategy as for G3BP1 protein interactors, we next overlapped data from the three above comparisons (G3BP1-APEX-ARS/G3BP1-APEX-UT; G3BP1-APEX-ARS/CTRL-APEX-ARS and G3BP1-APEX-UT/CTRL-APEX-UT) in a Venn diagram (Figure 5D). This defined four groups of transcripts including stress-dependent (482 transcripts; Group A), stress-sensitive (735 transcripts; Group B), stress-independent (175 transcripts; Group C) and transcripts with reduced G3BP1 association (dissociated/reduced; 1212 transcripts; Group D). The list of transcripts in each group is provided in the Supplementary Table S7. Raw data from APEX RNAseq experiments were submitted to NCBI GEO (https://www.ncbi.nlm.nih.gov/geo/query/acc.cgi?acc=GSE138058).

### Validation of G3BP1-associated transcripts

**Figure 6.**
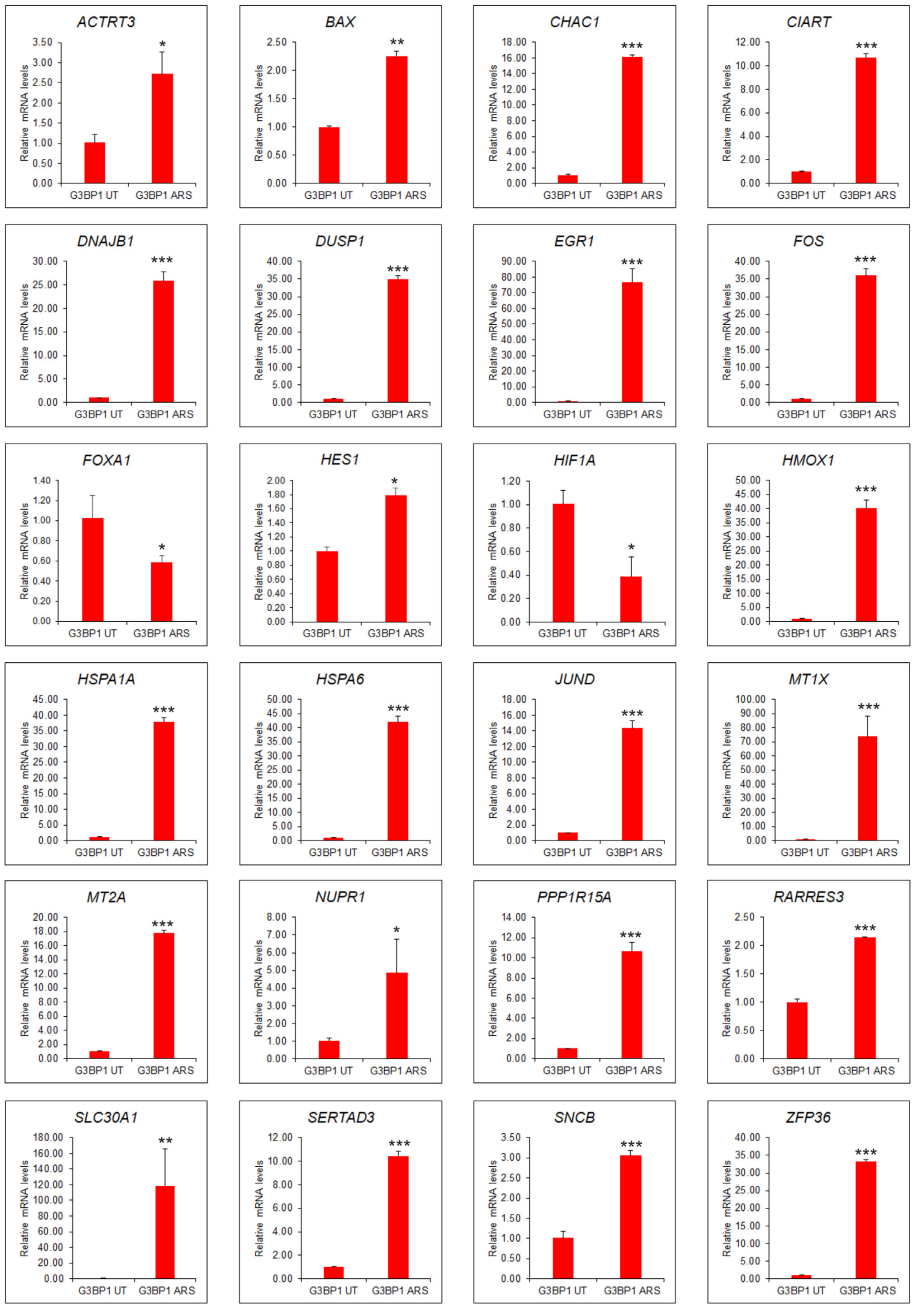
Validation of G3BP1-associated transcripts. Selected G3BP1-associated mRNAs extracted from vehicle treated or arsenite treated cells were subjected to qRT-PCR using primers specific to the transcripts as indicated in the figure. Mean values ± SD are shown for three independent experiments. ***P < 0.001; **P < 0.01; *P < 0.05.

To validate this approach, we used qRT-PCR to confirm selected transcripts purified as above from APEX-G3BP1-UT and APEX-G3BP1-ARS lysates. Purified RNA was reverse transcribed and subjected to qRT-PCR using primers for selected transcripts. *ACTRT3, BAX, CHAC1, CIART, DNAJB1, DUSP1, EGR1, FOS, HES1, HMOX1, HSPA1A, HSPA6, JUND, MT1X, MT2A, NUPR1, PPP1R15A*,*RARRES3, SLC30A1, SERTAD3, SNCB* and *ZFP36* transcripts were significantly associated with G3BP1 in arsenite stressed compared to vehicle treated cells (Figure 6). In contrast, while *HIF1A* and *FOXA1* were G3BP1-associated in vehicle treated cells, this association was reduced under arsenite stress, consistent with our APEX studies. A caveat of our approach is that transcripts identified above may also associate with G3BP1 indirectly through other RNA binding proteins binding to G3BP1. To independently validate our data using an alternative strategy, we treated PC3 cells with arsenite versus vehicle alone (UT), and exposed cells to UV radiation to crosslink proteins and associated RNAs. Affinity purification with anti-G3BP1 antibodies was then used to pull-down associated RNAs, followed by qRT-PCR to detect selected transcripts, namely *CHAC1, CIART1, DNAJB1, DUSP1, EGR1, FOS, HMOX1, HSPA1A, HSPA6, JUND, MT1X, MT2A, PPP1R15A* and *SLC30A1*. As shown in Supplementary Figure S6, this demonstrated significantly increased G3BP1 association of each transcript under arsenite stress, in contrast to *HIF1A*, further validating the G3BP1-APEX data.

### G3BP1-associated transcripts and their partitioning to or from polysomes

**Figure 7.**
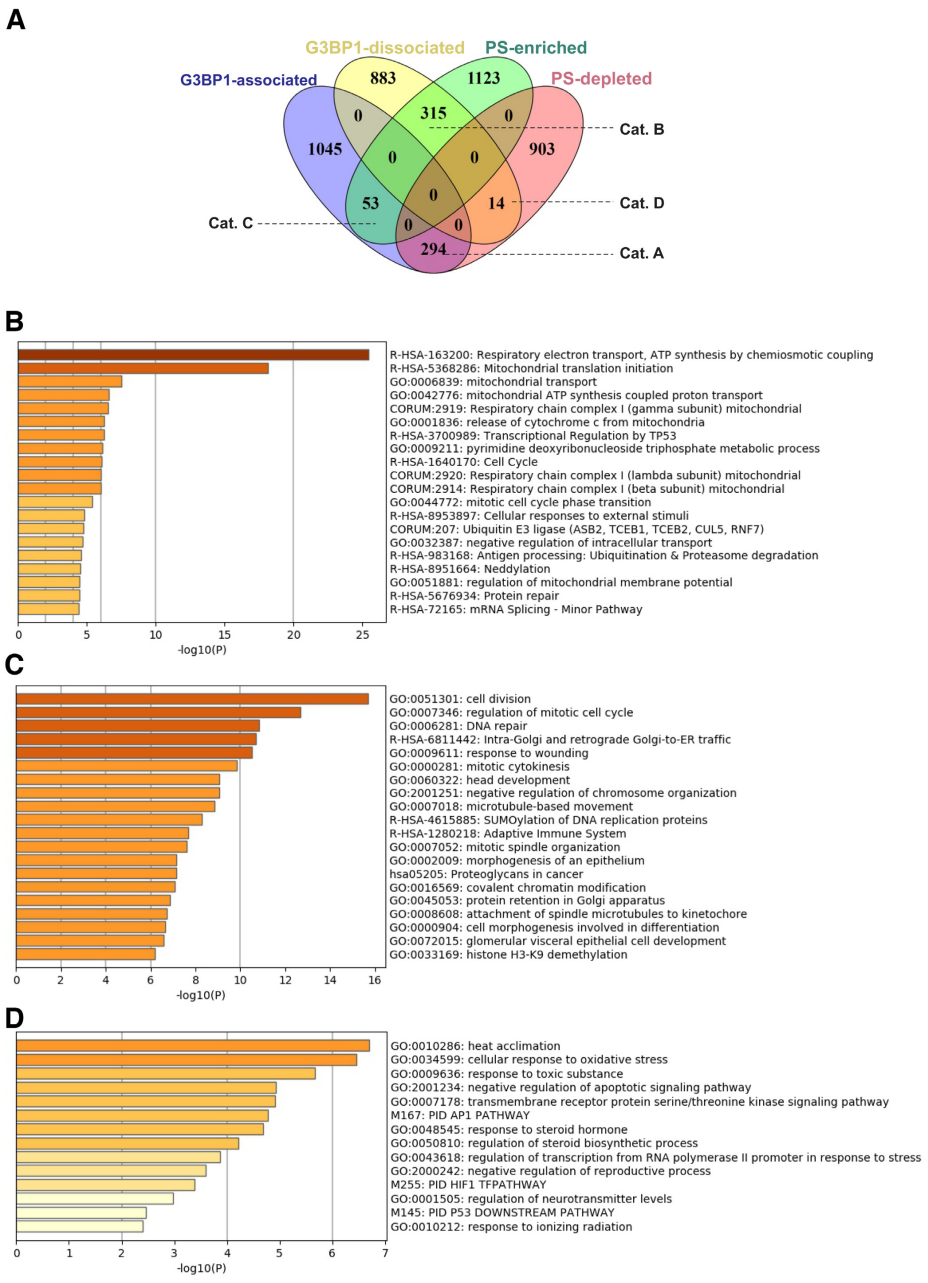
Compartmentalization of G3BP1-associated transcripts with PSs and their gene ontology analysis. (**A**) Venn diagram illustrating the compartmentalization of G3BP1-associated and dissociated/reduced transcripts within (PS-enriched) or away (PS-depleted) from PSs in stressed PC-3 cells revealed four categories; Cat. A-D. Gene ontology analysis of G3BP1 partitioned transcripts, (**B**) corresponding to Cat. A, (**C**) corresponding to Cat. B and (**D**) corresponding to Cat. C, using Metascape software.

We next wished to overlap the above data with the potential role of G3BP1 in partitioning mRNAs between PS and non-PS fractions under arsenite stress. To do this, we cross-referenced transcripts detected as either being G3BP1-associated (groups A–C from Figure 5D) or dissociated from G3BP1 (group D from Figure 5D), with those designated as PS-enriched or PS-depleted transcripts within the same samples (Figure 7A). From this comparison, we identified three major categories of transcripts, namely transcripts that were both associated with G3BP1 and PS-depleted (Category A; 294 transcripts), transcripts that were dissociated from or reduced in their association with G3BP1 and also PS-enriched (Category B; 315 transcripts) and transcripts that are both associated with G3BP1 and PS-enhanced (Category C; 53). There was also a minor category of 14 transcripts that were G3BP1-dissociated and PS-depleted (Category D) (Supplementary Table S8).

We next assessed specific cellular processes associated with transcripts from each of the major categories of Figure 7A (Categories A, B and C) using Metascape software. Ontology analysis of G3BP1-associated/PS-depleted transcripts (Category A) revealed correlations with mitochondrial functions including respiratory electron transport, mitochondrial translation, mitochondrial transport, release of cytochrome c from mitochondria, and mitochondrial ATP synthesis (Figure 7B). Examples of transcripts from this category that modulate these functions include *BNIP3*, *GPX1*, *ATP5I*, *ATPIF1, TSPO*, *BAX*, *CDK5*, *NDUFA1*, *NDUFA3*, *PDCD5* and *FAM162A*. Since mRNAs in this category were defined as being G3BP1-associated and PS-depleted, the prediction is that mitochondrial functions such as respiration and ATP production are inhibited by arsenite stress. Accordingly, as described above, OCR and ATP production were significantly reduced in arsenite treated cells compared to vehicle alone (Figure 2B and D), supporting the ontology analysis. Moreover, arsenite treatment was previously reported to reduce mitochondrial bioenergetics (49,50). BAX functional studies are included in the next section.

Similar analysis of G3BP1-dissociated and PS-enriched transcripts (Category B of Figure 7A) revealed links to cell cycle related processes, including cell division, regulation of mitotic cell cycle, DNA repair, mitotic cytokinesis, predicting that cell division and proliferation are blocked under arsenite stress and that consequent stress responses are activated (Figure 7C). Examples that modulate cell cycle and wound healing functions include *APC*, *BUB1B*, *CENPE*, *CENPF*, *CLTC*, *DYNC1H1*, *ECT2*, *INCENP*, *KIF11*, *MYH10*, *USP9X*, *SMC1A*, *TNKS*, *HIF1A*, *EGFR*, *MTOR*, *NOTCH1* and *NOTCH2*. Accordingly, arsenite blocked cell cycle progression (see Figure 2E), in keeping with previous reports that arsenite stress induces cell cycle arrest at the G2/M transition (54,58). HIF1α functional studies are included in the next section.

Finally, biological processes significantly correlated with G3BP1-associated and PS-enriched transcripts (Category C of Figure 7A) are mainly involved in stress response pathways such as heat acclimation, cellular responses to oxidative stress, toxic substance, and negative regulation of apoptosis (Figure 7D). Examples of transcripts that modulate the above pathways include *HSPA1A*, *FOS*, *ADM*, *HMOX1*, *HSPA1B* and *HSPA6*. This suggests that encoded proteins in this category are stress response factors, such as HSP70, and aligns with our previous work that G3BP1 is cytoprotective and pro-metastatic under different stress conditions (20).

### Effects of G3BP1-associated transcript partitioning on protein expression

**Figure 8.**
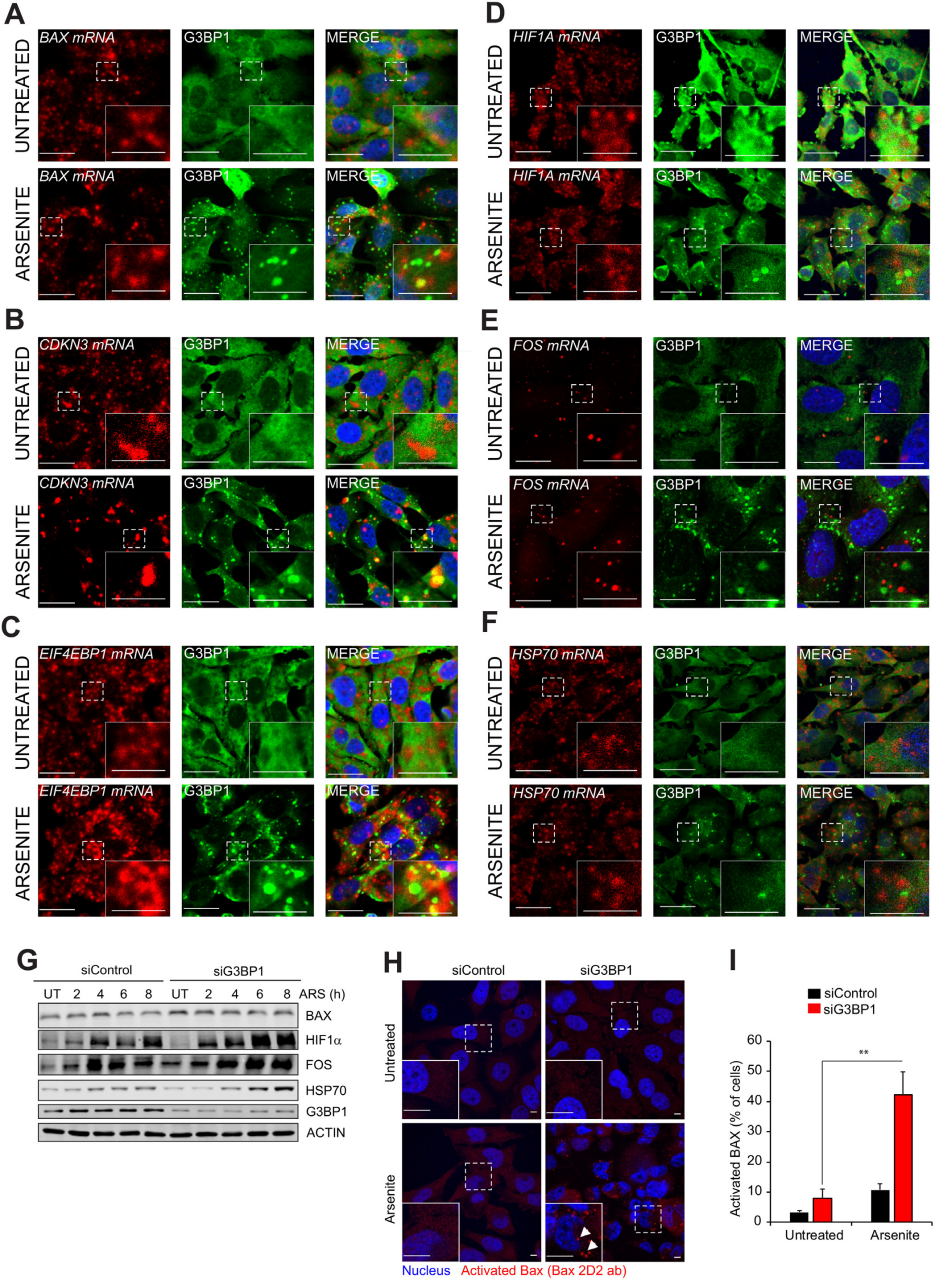
Protein expression and localisation of G3BP1-partitioned transcripts. (**A–F**) RNA *in situ* hybridization of different G3BP1-partitioned transcripts using mRNA probes as indicated (red panels). The *in-situ* slides were subjected to IF staining using anti-G3BP1 antibodies (green panels) to detect colocalization with G3BP1 in presence and absence of stress. A portion of each main figure panel is enlarged and shown as an insert. Scale, 10 um. (**G**) Western blot analysis of selected G3BP1-partitioned transcripts (see Materials and Methods for details). (**H**) Activated BAX detected using anti-BAX (2D2) antibodies in untreated or arsenite treated cells. A portion of each main figure panel is enlarged and shown as an insert. White arrows point to the spots representing activated BAX. Scale, 10 um. (**I**) Quantification of BAX activation. Mean values ± SD are shown for three independent experiments. **P < 0.01.

We next explored the functional consequences of G3BP1-mediated transcript partitioning on protein expression in each of the above transcript categories of Figure 7A under stress. To do this, we tested if G3BP1 depletion affects levels of selected proteins encoded by representative transcripts from each category (i.e. G3BP1-associated/PS-depleted, G3BP1-dissociated/PS-enriched, and G3BP1-associated/PS-enriched transcripts). PC-3 cells transfected with two independent siRNAs, each effecting >90% G3BP1 kd (Supplementary Figure S7A), were treated with arsenite as above. G3BP1-associated/PS-depleted transcripts (Category A) that became PS-depleted by arsenite treatment are predicted to sequester to G3BP1-nucleated SGs under stress. An example is the *BAX* pro-apoptotic BCL-2 family member (Supplementary Table S8). By RNA *in situ* hybridization, *BAX* mRNA was at least partially recruited to SGs under arsenite treatment (Figure 8A; quantified in Supplementary Figure S7B). While BAX protein expression was reduced by arsenite treatment in control cells (Figure 8G, left lanes), its expression was restored by G3BP1 kd under the same condition (Figure 8G, right lanes), consistent with the notion that *BAX* transcripts are no longer recruited to SGs in the absence of G3BP1, and therefore become available for translation. BAX activation in G3BP1 kd cells was confirmed using activation-specific 2D2 antibodies (Figures 8H and I) (59). *CDKN3* and *EIF4EBP1* are additional examples of transcripts in this sub-group, and accordingly, each of these transcripts is recruited to SGs after arsenite stress (Figure 8B and C; quantified in Supplementary Figure S7B).

G3BP1-dissociated/PS-enriched (Category B) transcripts represent mRNAs bound by G3BP1 under non-stressed conditions, but which dissociate from G3BP1 during stress. An important example is *HIF1A*, which encodes HIF1α, a well known pro-oncogenic driver (60). There was no significant difference in overall *HIF1A* transcripts in arsenite versus vehicle treated cells (Supplementary Figure S7C). However, under arsenite treatment, *HIF1A* binding to G3BP1 was reduced (Figure 6; see *HIF1A* panel), while its association with PSs increased (Figure 1E). *HIF1A* was excluded from SGs in stressed cells by RNA *in situ* hybridization (Figure 8D; quantified in Supplementary Figure S7B). Moreover, G3BP1 kd enhanced HIF1α protein expression under arsenite (Figure 8G; right lanes). Collectively, this data is consistent with increased availability of *HIF1A* transcripts for translation under arsenite stress. Therefore translation of at least some mRNAs from each of the above categories appears to be regulated by G3BP1, either by release from (e.g. *HIF1A*) or sequestration by G3BP1 (e.g. *BAX, CDKN3* and *EIF4EBP1*) under arsenite stress.

For Category C transcripts (i.e. G3BP1-associated and PS-enriched under arsenite treatment), examples include *FOS* and *HSP1A1*. Corresponding proteins were enhanced by arsenite stress (Figure 8G, left lanes), consistent with increased expression of *FOS* and *HSP1A1* mRNAs in the presence of arsenite and their enrichment in PSs (Supplementary Table S1; Figure 1E). Unexpectedly, however, FOS and HSP70 proteins were further increased by G3BP1 kd (Figure 8; right lanes. This suggests that G3BP1 inhibited their mRNA translation under arsenite treatment, even though these transcripts were enriched in PSs). This was not due to colocalization in G3BP1-containing SGs (Figure 8E and F), pointing to a G3BP1-linked repressive process other than via SG sequestration. Further studies are necessary to elucidate this as-yet unknown mechanism. Together, these studies highlight G3BP1-dependent regulation of translation of different classes of transcripts. Together, these results strongly point to a key role for G3BP1 as a cytoprotective factor in prostate cancer cells, likely through its effects on partitioning of transcripts between different mRNA compartments for activation or suppression of their translation.

## DISCUSSION

Stress-induced repression of global protein synthesis is accompanied by selective translation of mRNAs encoding proteins that are vital for stress recovery, but how these two processes are coordinated is not well understood. While SG formation has been proposed as a driver of stress-induced translational repression (61), more recent studies have questioned this view, mainly because only ∼10% of messages are sequestered to SGs under stress (30). Moreover, few studies have examined the partitioning of transcripts between SGs, RBPs and PSs under stress, and how this allows cells to repress pro-cell death proteins and selectively synthesize cytoprotective proteins during stress adaptation. Here we adopted a dual approach to characterize stress-induced changes in transcript partitioning between the SG-associated RBP, G3BP1 and the polysomal compartment in PC-3 cells to gain insights into translational reprogramming under stress. Sucrose gradient polysomal fractionation (SGPF) (32,33) and RNAseq was used to define selective enrichment or depletion of transcripts in PSs under arsenite stress. In parallel, APEX-affinity tagging (27,34,57) was used to profile transcripts and proteins interacting with G3BP1 under arsenite stress. PS- and G3BP1-associated transcripts were then compared to define roles for G3BP1 in stress-induced translational reprogramming. We found that oxidative stress profoundly altered the partitioning of transcripts between these compartments. Under arsenite stress, G3BP1-associated transcripts tended to be PS-depleted and encoded proteins involved in mitochondrial bioenergetics and cytoprotection. PS-enriched transcripts that disassociated from G3BP1 under stress broadly encoded proteins involved in cell cycle regulation, and transcripts that were both G3BP1-associated and enriched in PSs encoded proteins involved in diverse stress response pathways, including heat shock responses. Therefore, G3BP1-mediated transcript partitioning reprograms mRNA translation to support stress adaptation and cell survival.

Among transcripts that were differentially associated with G3BP1 and also PS-depleted under arsenite stress were those encoding proteins involved in mitochondrial functions (e.g. metabolic enzymes involved in respiration and oxidative phosphorylation) as well as pro-apoptotic factors (e.g. BAX, BAD, BID). We demonstrated that a number of G3BP1-associated/PS-depleted transcripts also segregated to SGs under stress, suggesting that G3BP1 sequesters certain transcripts away from PSs to SGs to block their translation in stressed cells. This is predicted to reduce corresponding protein synthesis, such as to support cytoprotective shifts in bioenergetics and to inhibit apoptosis, respectively, in stressed tumor cells. This was supported by our functional studies, which demonstrated that OCR and ECAR as well as ATP production were significantly reduced in arsenite treated cells, aligning with published data showing that arsenite reduces mitochondrial bioenergetics (49,50). A specific example of a pro-apoptotic factor in this category is BAX (which is also a mitochondrial associated protein). We found that BAX expression and activation were both reduced by arsenite stress, and that *BAX* transcripts were PS-depleted and associated with SGs under arsenite. Notably, G3BP1 kd increased both protein levels and BAX activation, consistent with *BAX* transcripts no longer being recruited to SGs in the absence of G3BP1, and therefore available for translation. These observations highlight potential mechanisms of stress-induced selective translation by which cancer cells co-opt homeostatic stress responses to support cell survival (such as by reducing pro-apoptotic protein expression) and potentially therapy resistance, although further studies are required to specify which translationally regulated pathways are most critical for cytoprotection under oxidative stress.

Another group of transcripts comprised those that were G3BP1-associated under non-stress conditions, but were reduced their association with G3BP1 and enriched in the PSs in the presence of arsenite. This category included transcripts involved in multiple aspects of cell cycle regulation and wound healing, suggesting that G3BP1 functions to translationally silence these transcripts until cells are exposed to stress. We observed that cell cycle progression was repressed by arsenite treatment, consistent with previous reports that arsenite induces cell cycle arrest at the G2/M transition (54,58). Therefore we speculate that transcripts in this group encode cell cycle inhibitory elements, although further studies are necessary to uncover the details of this process. Another example in this category is *HIF1A*. This transcript became PS-enriched and was excluded from SGs under arsenite, and HIF1α protein expression was enhanced under this condition. Notably, G3BP1 kd actually increased HIF1α protein expression under oxidative stress, pointing to a previously unexplored link between G3BP1 and HIF1α signalling. Moreover, in addition to being a hypoxia-induced cytoprotective protein, HIF1α is also known to induce cell cycle arrest (62,63). Therefore an interesting possibility requiring further investigation is that arsenite-induced cell cycle arrest is in part be mediated via dissociation of *HIF1A* from G3BP1 to increase its translation under stress.

We also observed a third category comprised of transcripts that were G3BP1-associated but PS-enriched under arsenite stress. Included in this group are transcripts encoding stress responsive elements, including HSP70 heat shock proteins and survival factors such as FOS. Since these transcripts were PS-enriched by arsenite stress and excluded from SGs, it is predicted that expression of encoded proteins in this class are enhanced under stress. However, we found that both FOS and HSP70 levels were actually increased by G3BP1. An intriguing possibility is that G3BP1 inhibits their mRNA translation under stress, even while transcripts are present in polysomes. While difficult to explain and requiring further studies, such a process could limit uncontrolled translation of specific transcripts that might otherwise be detrimental to tumor cells.

Several recent studies have reported SG proteomes and transcriptomes, each using different stress conditions, cell lines, and analytic technologies (18,27,30,31). Although our strategy was specifically designed to identify G3BP1-associated proteins and transcripts, rather than those localized exclusively to SGs, we compared our data with SG proteomes and transcriptomes from the above studies, as well as with publically available RNA granule/SG databases (http://rnagranuledb.lunenfeld.ca; https://msgp.pt/). At the proteomic levels, our studies detected many previously catalogued G3BP1/SG associated proteins (132 proteins; see Figure 4B). This included TDP-43, RACK1, VCP, HNRNPK, PFN1, RNH1, SERBP1 and SND1, which were not detected in the G3BP1-APEX studies conducted by (18) performed using HEK293T and NPC cells, highlighting the potential variability in such studies. Since SGs are estimated to contain only ∼20% of cellular G3BP1 under arsenite stress (9), it is not surprising that SG proteomes show incomplete overlap with more global G3BP1-associated proteomes. When we compared G3BP1-associated transcripts to two recently published SG-transcriptomes (30,31), we found only 2% similarity of our G3BP1-associated mRNAs with the first study, which was conducted using arsenite treatment using U2OS osteosarcoma cells (30). Compared to the second study (31) using HEK293 cells, we observed 38% similarity of G3BP1-associated mRNAs under arsenite stress, 32% similarity under heat shock and 2% similarity under ER stress. Extensive research indicates that multiple factors regulate the recruitment of mRNPs to RNA granules, including dynamic, stable and extended interactions affected by mRNA translation status, length, and efficiency as well as granule size, that collectively regulate RNP granule dynamics (30,64). Moreover, each study used a different methodology to isolate SGs, as well as different cell lines, treatment times and concentrations of arsenite to capture what is an extremely dynamic process. Therefore it is perhaps not surprising that results from different studies yield some degree of non-overlapping data.

In summary, our studies reveal different categories of stress-regulated transcripts in the context of G3BP1. First are transcripts that are G3BP1-associated and are PS-depleted, at least some of which are translationally repressed by G3BP1 under oxidative stress. Another class of transcripts bind G3BP1 under ambient conditions, but are released for rapid translation in PSs in response to oxidative stress. Such transcripts may be directly regulated by G3BP1, and G3BP1 depletion enhances their translation. Finally, we found that some transcripts are associated with G3BP1 and also PS-enriched under stress, although exactly how this regulates their translation requires further analysis. These categories illustrate that G3BP1 can regulate the translation of distinct classes of transcripts. Whether such functions are co-regulated by the other major G3BP isoform, G3BP2, or by other RBPs that are in complex with G3BP1 under stress, is unknown. G3BP1 therefore appears to play a key role in selective translation by regulating transcript trafficking to and from PSs to influence the activity of stress adaptive survival pathways. Whether similar modes of regulation occur under other stresses, such as hypoxia, remains an important open question.

## DATA AVAILABILITY

RNA-seq data reported in this article is available in GEO under accession number GSE138058. Mass spectrometry data provided in this article is available in PRIDE under project accession PXD015491.

## Supplementary Material

gkaa376_Supplemental_Files
