# Supplementary material for: G3BP1-linked mRNA partitioning supports selective protein synthesis in response to oxidative stress
Source: Nucleic Acids Res. 2020 May 14;48(12):6855–73. doi: 10.1093/nar/gkaa376 (PMC7337521; doi:10.1093/nar/gkaa376)
Supplement: gkaa376_Supplemental_Files [file gkaa376_supplemental_files.zip › NAR-03218-V-2019 .R1_Supplementary Figure and Table Legends.docx]

**SUPPLEMENTARY FIGURE LEGENDS**

**Fig. S1. Analysis of PSseq and total RNAseq. (A)** Heat map on the differential expression analysis and (**B**) correlation analysis of transcripts in the PSseq of vehicle treated and arsenite treated cells. (**C**) Heat map on the differential expression analysis and (**D**) correlation analysis of transcripts in the total RNAseq of vehicle treated and arsenite treated cells.

**Fig. S2.** **Newly synthesized proteins in vehicle treated and arsenite stressed cells.** (**A**) Newly synthesized proteins in untreated and arsenite treated cells were detected by Western blotting (see Methods for details) (**B**) Venn diagram that compares PS-enriched, PS-depleted and non-significant transcripts i.e. PS-unchanged, transcripts that are allocated as neither enriched nor depleted in PSseq analysis, with 362 proteins that evade translational repression after arsenite stress ([41](#_ENREF_41)).

**Fig. S3. Correlation analysis of G3BP1-associated proteins.** Correlation analysis of G3BP1-APEX-ARS/G3BP1-APEX-UT (**A**), G3BP1-APEX-ARS/CTRL-APEX-ARS (**B**) and G3BP1-APEX-UT/CTRL-APEX-UT (**C**).

**Fig. S4. Validation of G3BP1 associated proteins. (A)** Association of CNOT1 with G3BP1 is reduced after arsenite stress. (**B**) Detection of G3BP1-associated proteins in untreated and arsenite treated cell by ribo-imunoprecipitation using anti-G3BP1 antibodies (RIP) and Western blotting.

**Fig. S5. Cufflinks analysis of G3BP1-associated transcripts.** Heat map on the differential expression and correlation analysis of triplicate samples that compares G3BP1-APEX-ARS/G3BP1-APEX-UT (**A, B**), G3BP1-APEX-ARS/CTRL-APEX-ARS (**C, D**) and G3BP1-APEX-UT/CTRL-APEX-UT (**E, F**).

**Fig. S6. Detection of G3BP1 associated transcripts by qPCR.** PC-3 cells vehicle treated or treated with arsenite were subjected to cross-linking using UV-radiation. The cell lysates were subjected to ribo-immunoprecipitation (RIP) using anti-G3BP1 antibodies. G3BP1-associated RNAs were extracted and subjected to qRT-PCR using primers for a selected number of G3BP1-associated mRNAs from APEX studies. Mean values ± SD are shown for three independent experiments. ***p < 0.001; **p < 0.01; *p < 0.01.

**Fig. S7. (A)** **Knockdown of G3BP1 using two independent siRNAs.** PC-3 cells were transfected with two independent siRNAs targeting G3BP1. The cells were harvested after 3-days of transfection and the cell lysates were subjected to Western blotting using anti-G3BP1 and anti-ACTIN antibodies. Note that both siRNAs reduced G3BP1 levels to >90%. (**B**) **Quantification of cells that shows co-localisation of different transcripts with G3BP1 after arsenite stress.** Mean values ± SD are shown for five independent experiments. (**C**) ***HIF1A* mRNA levels were measured by qRT-PCR using total RNA extracted from vehicle treated or arsenite treated cells.** Mean values ± SD are shown for three independent experiments. ns, non-significant.

**SUPPLEMENTARY TABLE LEGENDS**

**Table S1. PSseq data from vehicle treated and arsenite treated PC-3 cells.** PS-enriched transcripts were defined as log2fc of arsenite treated/vehicle treated is 1 and above, and PS-depleted transcripts were defined as log2fc of arsenite treated/vehicle treated is -1 and below, both with p values = <0.05.

**Table S2.** **Total RNAseq data from vehicle treated and arsenite treated cells.** Total transcripts upregulated after arsenite stress were defined as log2fc of arsenite treated/vehicle treated is 1 and above and total transcripts downregulated after arsenite stress were defined as log2fc of arsenite treated/vehicle treated -1 and below, both with p values = <0.05.

**Table S3. G3BP1-associated proteins.** Normalised abundance values of proteins and different comparisons - G3BP1-APEX-ARS/G3BP1-APEX-UT, G3BP1-APEX-ARS/CTRL-APEX-ARS, and G3BP1-APEX-UT/CTRL-APEX-UT are provided.

**Table S4. G3BP1-associated transcripts- G3BP1-APEX-ARS/G3BP1-APEX-UT.**

**Table S5. G3BP1-associated transcripts- G3BP1-APEX-ARS/CTRL-APEX-ARS.**

**Table S6. G3BP1-associated transcripts- G3BP1-APEX-UT/CTRL-APEX-UT.**

**Table S7. G3BP1-associated mRNAs as different categories from Fig. 5D.**

**Table S8. G3BP1-associated mRNAs partitioned with PSs from Fig. 7.**

**Table S9. Oligonucleotides used for qRT-PCR, siRNA transfections and RNA in situ hybridisation (ISH).**
