# Supplementary figures and images for: G3BP1-linked mRNA partitioning supports selective protein synthesis in response to oxidative stress
Source: Nucleic Acids Res. 2020 May 14;48(12):6855–73. doi: 10.1093/nar/gkaa376 (PMC7337521; doi:10.1093/nar/gkaa376)

Fig. S1

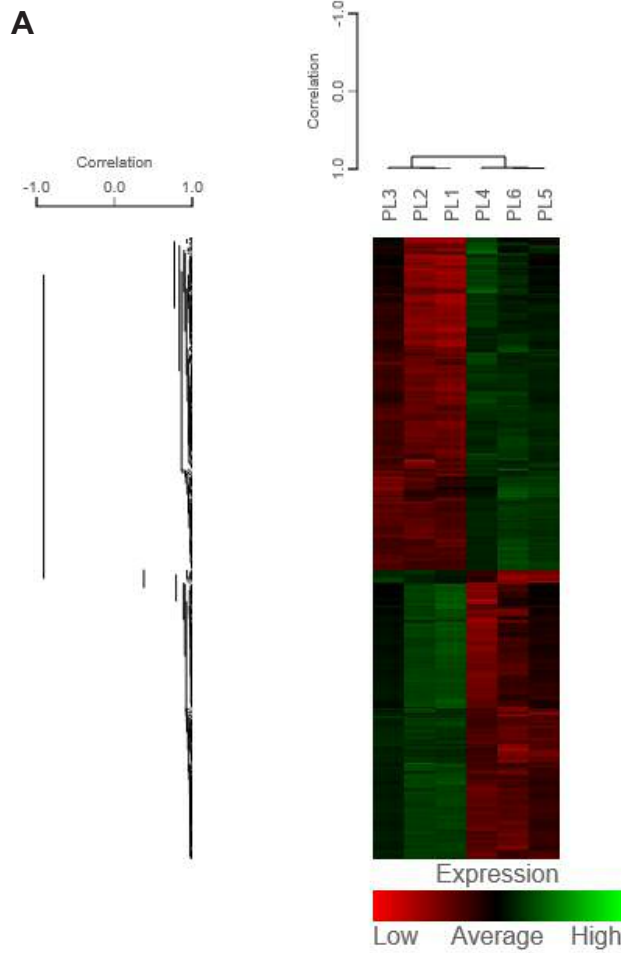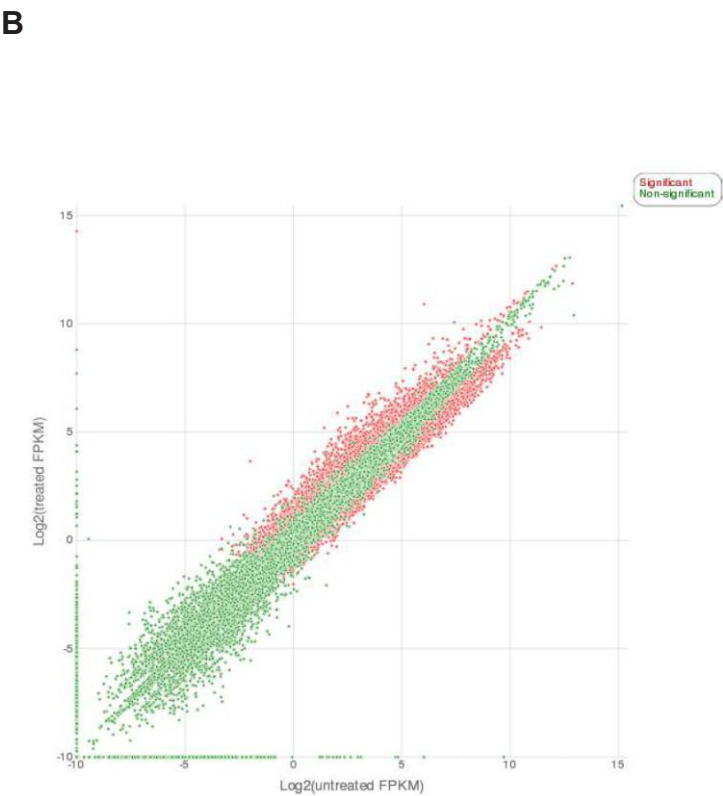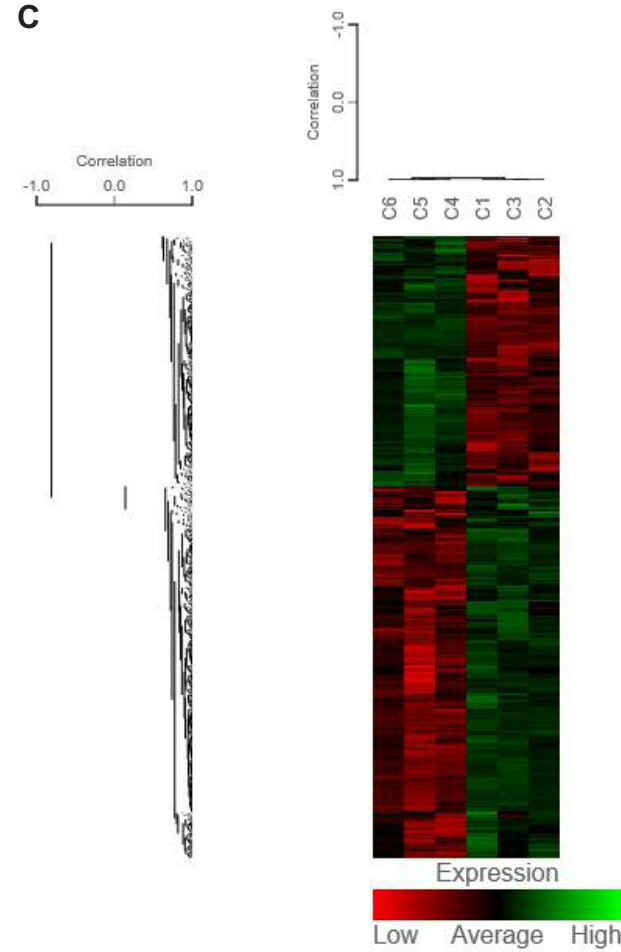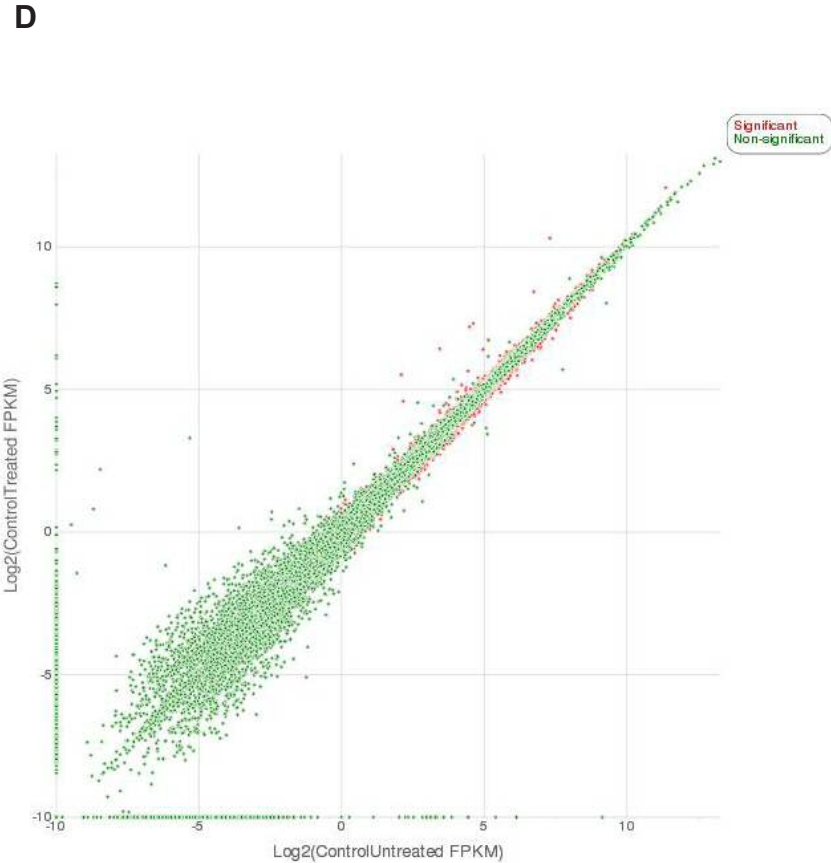

**Fig. S2**

**A**

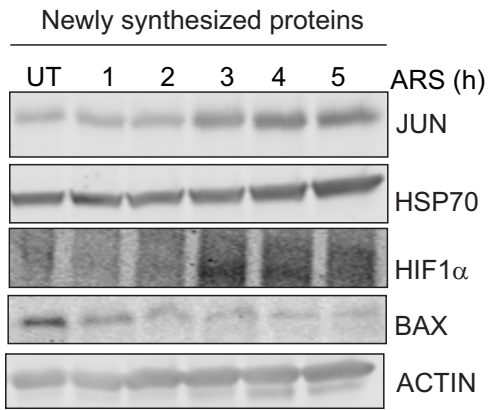

**B**

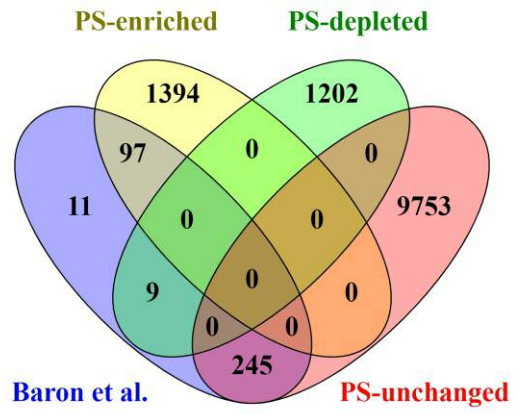

**Fig. S3**

**A**

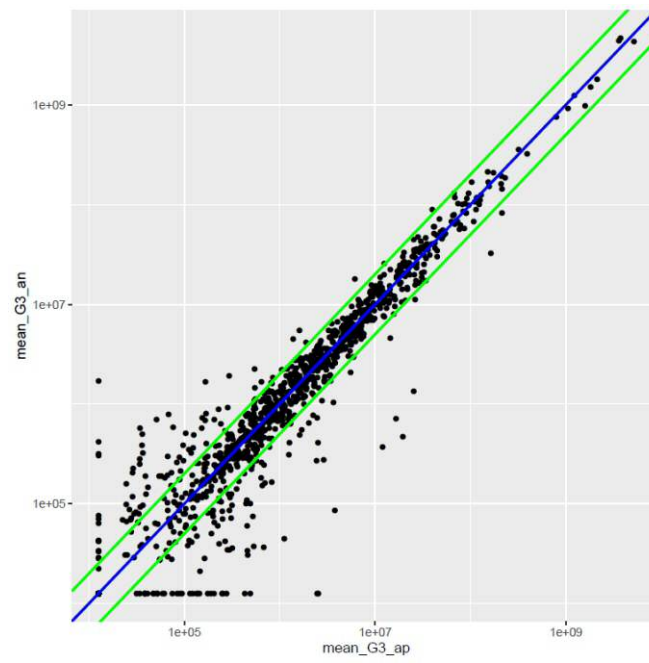

**B**

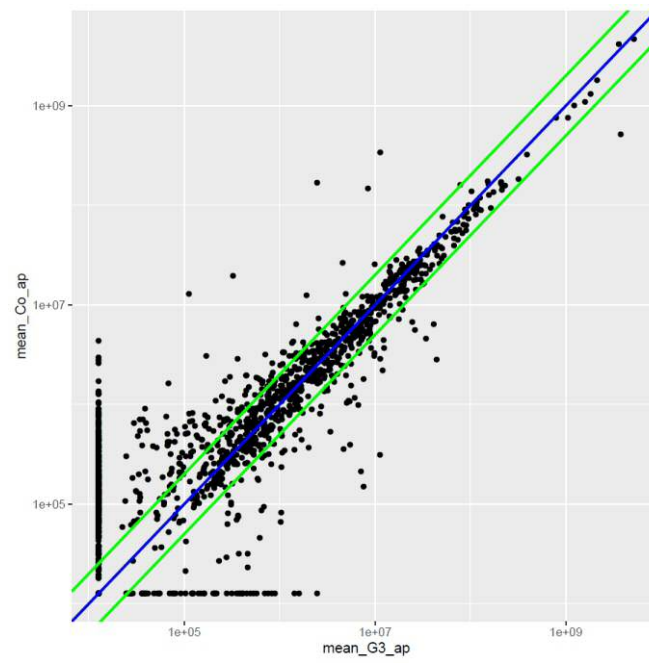

**C**

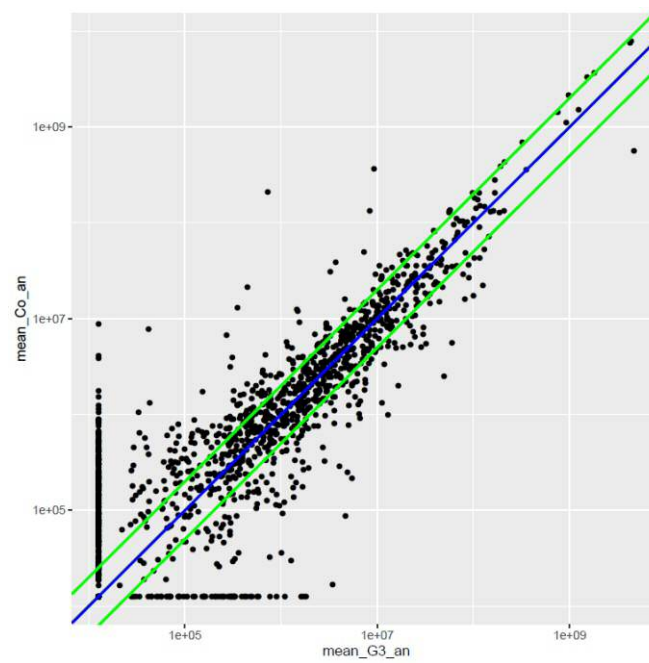

Fig. S4

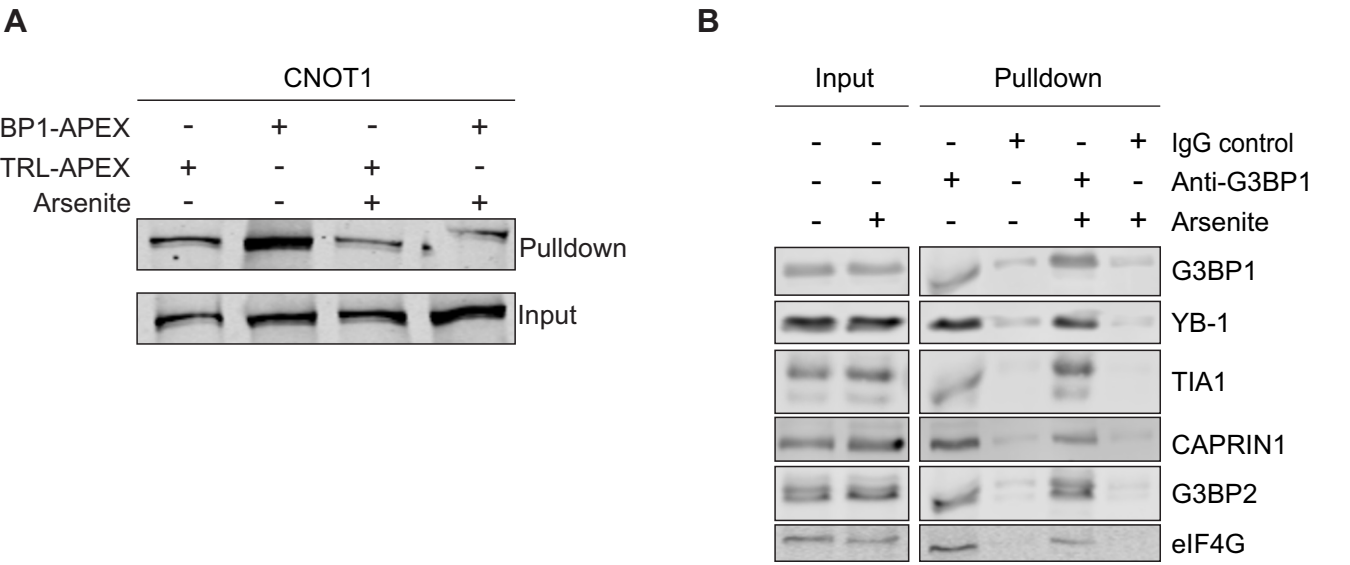

Fig. S5

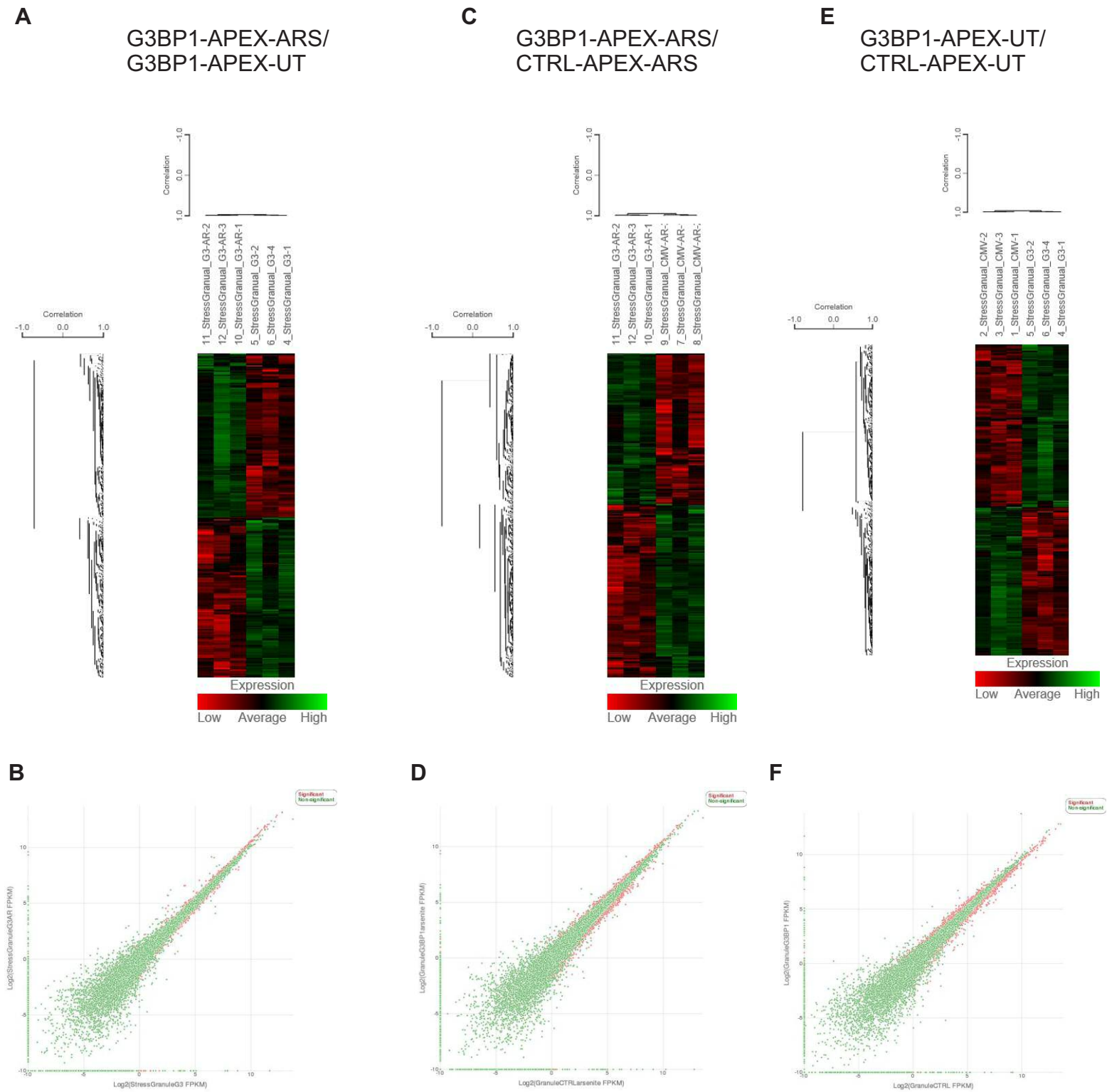

Fig. S6

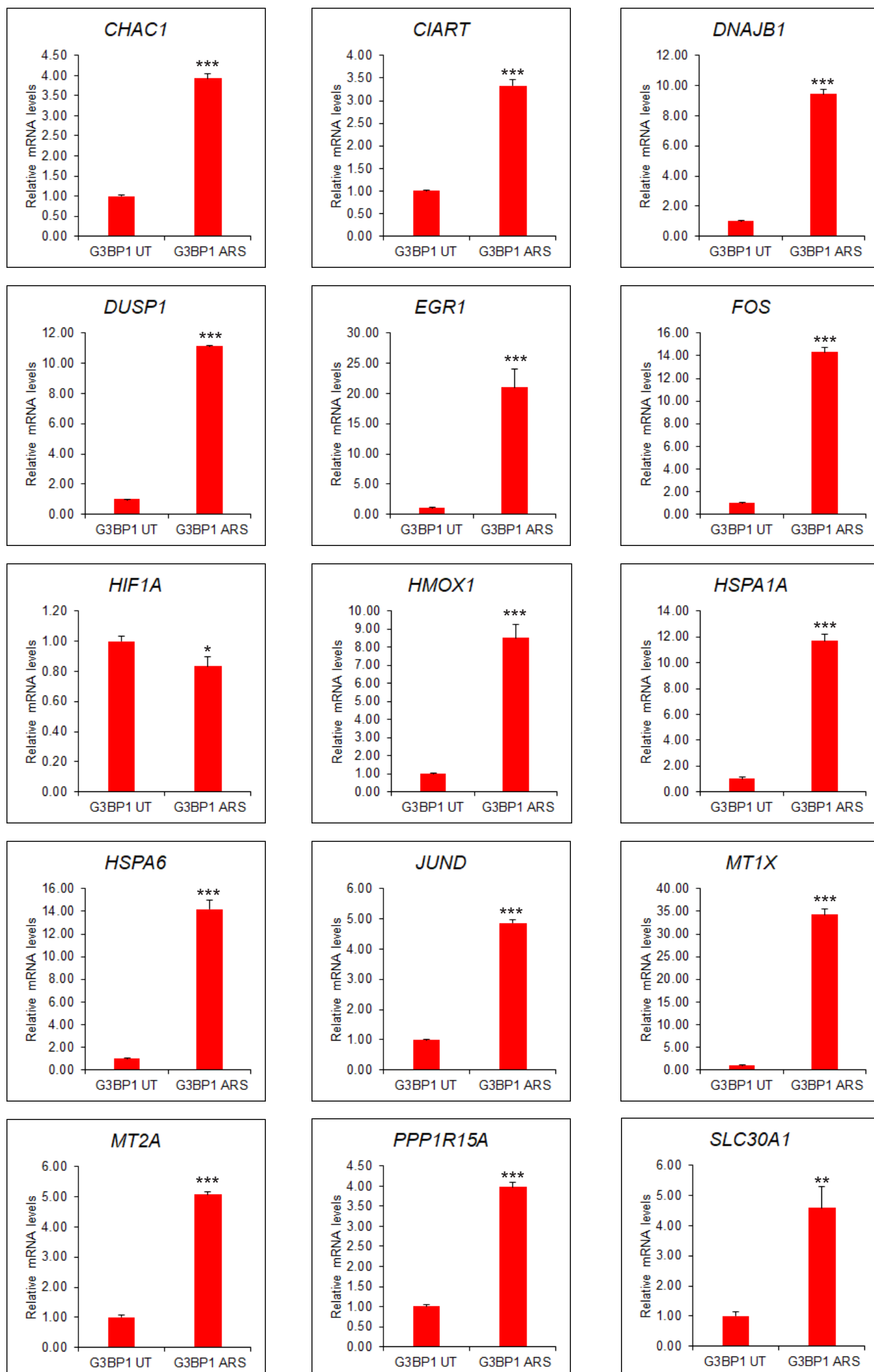

**Fig. S7**

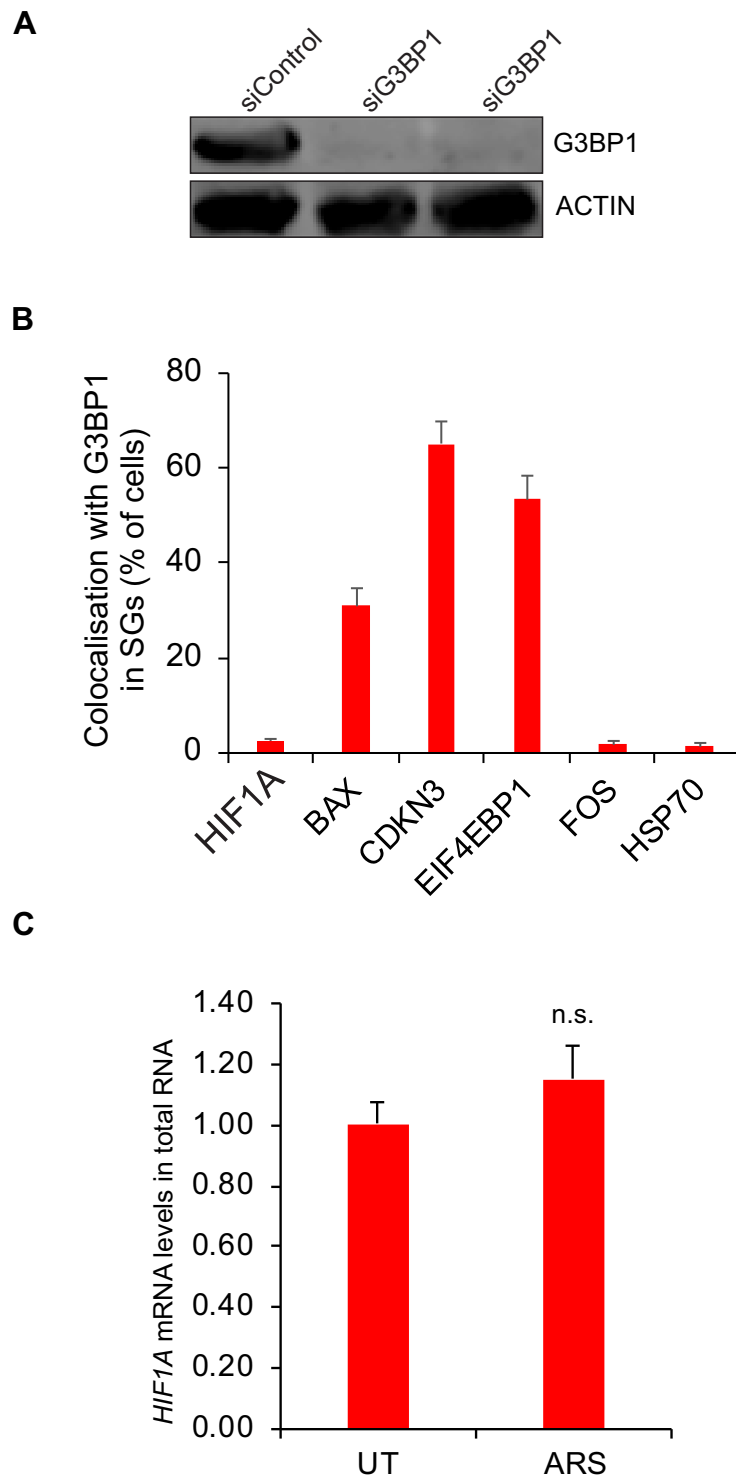

Supplement: gkaa376_Supplemental_Files [file gkaa376_supplemental_files.zip › NAR-03218-V-2019 .R1_Supplementary Figures.pdf]
